# Supplementary material for: Activation pathway of a G protein-coupled receptor uncovers conformational intermediates as targets for allosteric drug design
Source: Nat Commun. 2021 Aug 5;12:4721. doi: 10.1038/s41467-021-25020-9 (PMC8342441; doi:10.1038/s41467-021-25020-9)
Supplement: Supplementary file 1 — Supporting Information [file 41467_2021_25020_MOESM1_ESM.pdf]

Supporting Information

Activation pathway of a G protein-coupled receptor uncovers conformational intermediates as targets for allosteric drug design

Shaoyong Lu[1,2,✉,§], Xinheng He[3,4,§], Zhao Yang[5,§], Zongtao Chai[6,§], Shuhua Zhou[5,§], Junyan Wang[5], Ashfaq Ur Rehman[2], Duan Ni[2], Jun Pu[7], Jinpeng Sun[5,✉] and Jian Zhang[1,2,8,✉]

[1] College of Pharmacy, Ningxia Medical University, Yinchuan, Ningxia Hui Autonomous Region 750004, China

[2]State Key Laboratory of Oncogenes and Related Genes, Key Laboratory of Cell Differentiation and Apoptosis of Chinese Ministry of Education, Shanghai Jiao Tong University, School of Medicine, Shanghai 200025, China

[3]The CAS Key Laboratory of Receptor Research, Shanghai Institute of Material Medica, Chinese Academy of Sciences, Shanghai 201203, China

[4]University of Chinese Academy of Sciences, Beijing 100049, China

[5]Department of Biochemistry and Molecular Biology, Key Laboratory Experimental Teratology of Chinese Ministry of Education, School of Medicine, Shandong University, Jinan, Shandong 250012, China

[6]Department of Hepatic Surgery VI, Eastern Hepatobiliary Surgery Hospital, Second Military Medical University, Shanghai 200438, China

[7]Department of Cardiology, Renji Hospital, Shanghai Jiao Tong University, School of Medicine, Shanghai 200120, China

[8]School of Pharmaceutical Sciences, Zhengzhou University, Zhengzhou 450001, China

[§]These authors contributed equally to this work.

✉To whom correspondence should be addressed:

Dr. Jian Zhang; E-mail: jian.zhang@sjtu.edu.cn

Dr. Shaoyong Lu; E-mail: lushaoyong@sjtu.edu.cn

Dr. Jinpeng Sun; E-mail: sunjinpeng@sdu.edu.cn

Supplementary Note 1: Convergence Test for Free Energy Landscapes

Supplementary Note 2: Analysis of Reported Class A GPCR Structures

Supplementary Note 3: Unusual GPCR Structures on the Free Energy Landscape

Supplementary Note 4: Distribution of G protein-, β-arrestin-, and nanobody-bound GPCR Complex on the Free Energy Landscape

Supplementary Note 5: Validation of Markov State Model Based on the Activation Parameters

Supplementary Note 6: Protein Levels of AT_1 Receptor and Vasopressin 2 Receptor (V_2R)

Supplementary Note 7: Position of ECL2, TM6, and H8 in Different Macrostates

Supplementary Note 8: Secondary Structure of Different Macrostates

Supplementary Note 9: Community Dynamics and Signal Transmission of Each State

Supplementary Note 10: Choice of Atoms for the Measurement of Hydrophobic Network

Supplementary Note 11: Gaussian Accelerated MD Simulations for the Holo Systems

Supplementary Note 12: Intermediate-specific Interactions in the Other Two States

Supplementary Note 13: Protein Levels of WT AT_1 Receptor and Variants for the Intermediate-specific Micro-switches

Supplementary Note 14: WT AT_1 Receptor and Variants for the Intermediate-Specific Micro-switches

Supplementary Note 15: TICA Analysis of Trajectories

Supplementary Note 16: Construction of the AT_1 Receptor-Transducer System

Supplementary Note 17: AngII-induced Activation on WT AT_1 Receptor and Variants for Different tICA Macrostates

Supplementary Note 18: Pocket Location in the Three Macrostates

Supplementary Note 19: Molecular Docking towards P6 pocket

Supplementary Note 20: Clustered Mutation Experiment of P6 in the AT_1 Receptor

Supplementary Note 21: AlloSigMA Provides Guidelines for the Design of P6 Mutations

Supplementary Note 22: AngII-induced Activation on Wild Type AT_1 Receptor and Variants for P6

Supplementary Note 1: Convergence Test for Free Energy Landscapes

To confirm that the length of trajectories and the number of rounds are sufficient for a stable free energy landscape and transition pathway, we designed convergence tests via changing trajectory length and round number, respectively. In length test shown in Supplementary Fig. 1, we truncated the 150 trajectories into varied subsets and plotted corresponding free energy landscapes, namely 225 μs (1.5 μs*150, Supplementary Fig. 1A), 240 μs (1.6 μs*150, Supplementary Fig. 1B), 255 μs (1.7 μs*150, Supplementary Fig. 1C), 270 μs (1.8 μs*150, Supplementary Fig. 1D), 285 μs (1.9 μs*150, Supplementary Fig. 1E), and 300 μs (2.0 μs*150, Supplementary Fig. 1F). We also changed the rounds of simulation in Supplementary Fig. 2 and obtained free energy landscapes of 240 μs (2.0 μs*8*15, Supplementary Fig. 2A), 270 μs (2.0 μs*9*15, Supplementary Fig. 2B), and original 300 μs (2.0 μs*10*15, Supplementary Fig. 2C). With the same parameters, all subfigures show a highly similar appearance and conformational distribution, confirming that both the current trajectory timescale and simulation rounds are enough to explore the activation pathway of the AT_1 receptor.

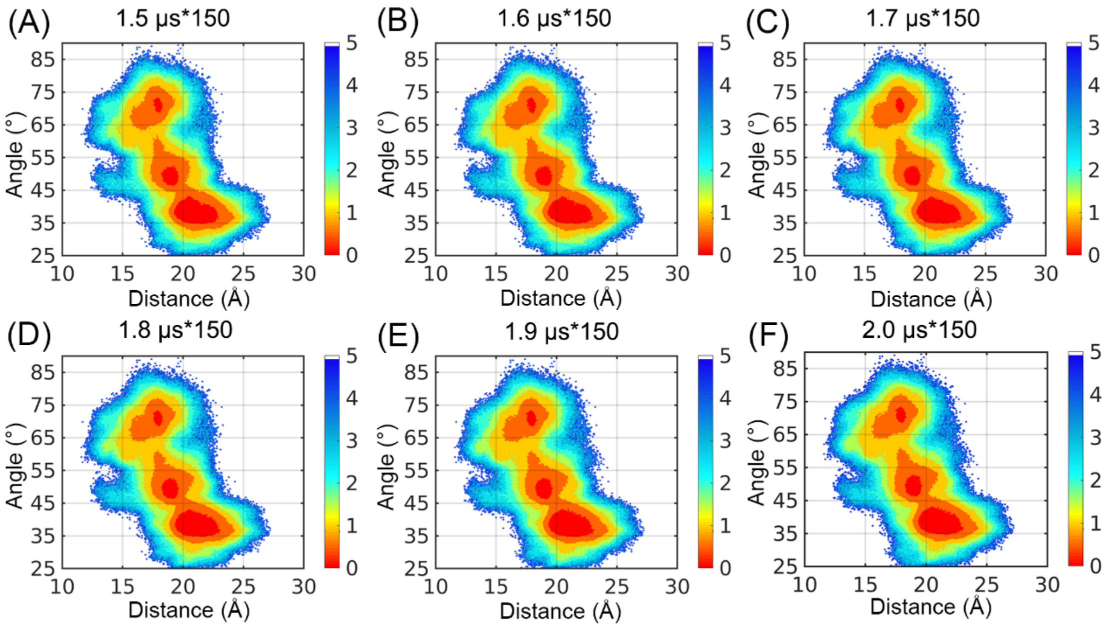

Supplementary Fig. 1: Free energy landscapes for different trajectory length (225 μs in (A), 240 μs in (B), 255 μs in (C), 270 μs in (D), 285 μs in (E), and 300 μs in (F)) in

the convergence test.

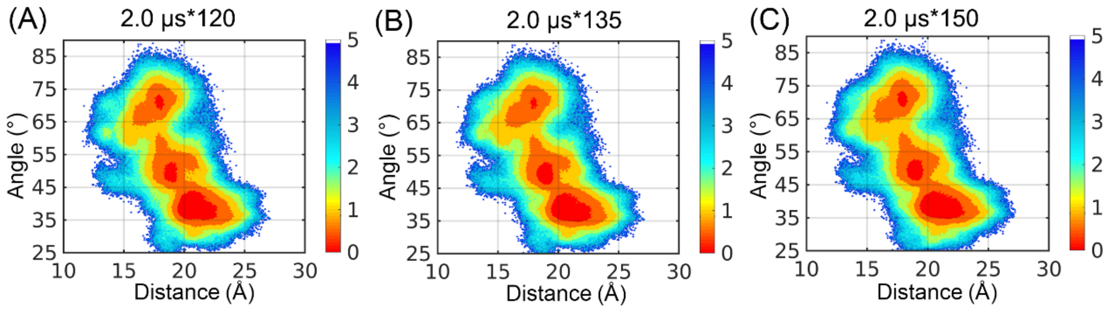

Supplementary Fig. 2: Free energy landscapes for different system rounds (8 rounds in (A), 9 rounds in (B), and 10 rounds in (C)) in the convergence test.

Supplementary Note 2: Analysis of Reported Class A GPCR Structures

To confirm the activation pathway of the AT_1 receptor discovered by our extensive MD simulations, we also collected currently published class A GPCR structures, measured their activation parameters (distance between $L^{5.55}$ and $N^{7.46}$; angle among $V^{2.41}$, $S^{6.47}$, and $F^{6.34}$), and projected them to the free energy landscape. Supplementary Data file 2 shows the statistical data of all collected GPCRs. We defined the receptor structures with inverse agonist- or antagonist-bound forms as inactive conformations, only agonist-bound forms as active conformations, and both agonist and G protein-, β-arrestin-, or nanobody-bound forms as fully active conformations.

Supplementary Note 3: Unusual GPCR Structures on the Free Energy Landscape

Several class A GPCRs have unique activation mechanisms. Thus, they are outliers in Fig. 2B and we show the specific structures in this Supplementary Note. As shown in Supplementary Fig. 3A, the inactive human platelet-activating factor receptor (PAFR) (PDB ID: 5ZKP) has a TM2 outward movement which increases the angle among 6.34, 6.47, and 2.41[1]. For the $P2Y_{12}R$ (PDB ID: 4NTJ) in Supplementary Fig. 3B, the pose of TM6 is distinctive. $N^{6.34}$ is on the edge of ICL3 and moves outwards, while $C^{6.47}$ becomes close to the center of TM bundles[2]. Thus, the activation angle is larger than other inactive structures.

In Supplementary Fig. 4, we show the comparison among two active CB_1R and one active CB_2R structures. CB_1R (PDB ID: 5XR8) and CB_2R (PDB ID: 6KPC) are GPCR-AM-841 complexes, while CB_1R (PDB ID: 6KQI) has both an agonist CP55940 and a negative allosteric modulator ORG27569 bound. 6KQI and 6KPC sample the same inactive-like conformation, whereas 5XR8 adopts an active-like conformation due to their different compositions.

As for the fully active structures, the most obvious outliers are rhodopsin structures (Supplementary Fig. 5). During activation. Rhodopsin receptor undergoes a limited TM5-TM7 inward movement and has a larger distance between residue positions at 5.55 and 7.46. Thus, they cluster at the right of the active cloud.

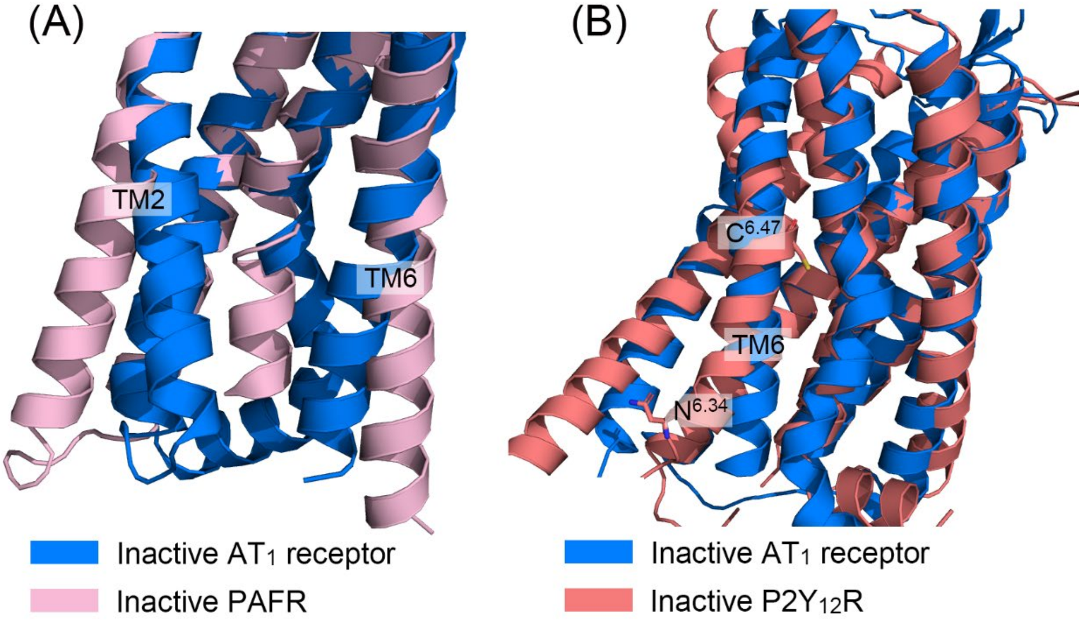

Supplementary Fig. 3: The outliers of inactive structures aligned to inactive AT_1 receptor. (A) Intracellular AT_1 receptor (blue) and PAFR (pink) are shown in cartoon. The unique relative position of TM2 is shown. (B) AT_1 receptor (blue) and $P2Y_{12}R$ (salmon) with distinct TM6 twist in its inactive state. Residues for angle measurement on TM6 are shown in sticks.

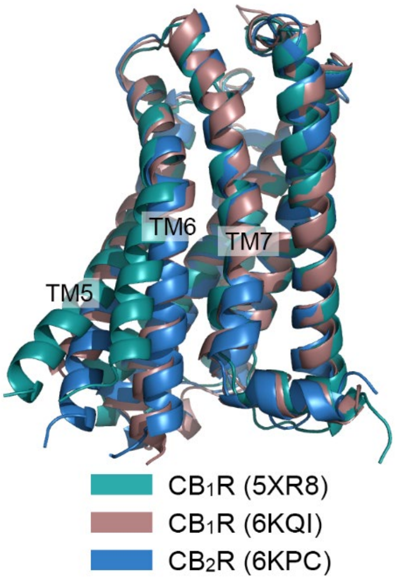

Supplementary Fig. 4: The outlier example of active structures, CBR. CB_1R-AM-841, CB_2R-AM-841, and CB_1R-CP55940-ORG27569 are depicted as green, brown, and blue cartoons, respectively.

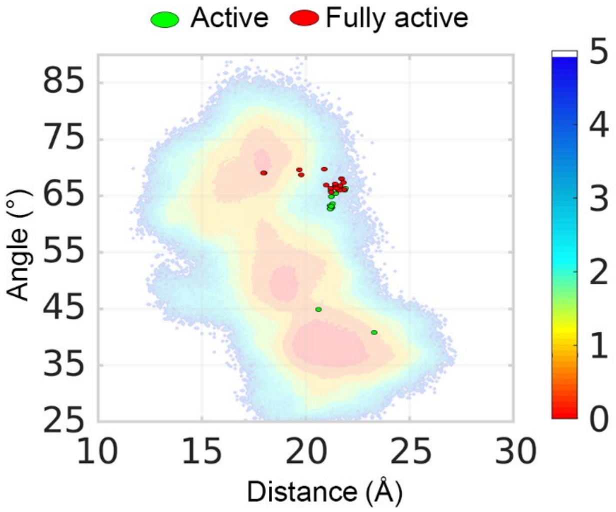

Supplementary Fig. 5: Projection of all reported fully active (red) and active (green) structures of rhodopsin onto the AT1 receptor conformational landscape. The unit of free-energy values is kcal/mol.

Supplementary Note 4: Distribution of G protein-, β-arrestin-, and nanobody-bound GPCR Complex on the Free Energy Landscape

We selected the G protein-, β-arrestin-, and nanobody-bound GPCR complexes from Supplementary Data file 1 and projected them on the free energy landscape (Supplementary Fig. 6). G protein-bound non-rhodopsin GPCR structures are mostly distributed in the active cloud, while the structures of rhodopsin-G protein complex situate at the right of the active basin. Gs protein has a bulkier α5 helix, which causes a larger TM6 movement of the receptors upon Gs binding than Gi/o. Thus, GPCR-Gs structures mostly cluster at the top right of the active cloud and GPCR-Gi/o structures lie in a lower position with a smaller angle. As for the TM5-TM7 movement, the distribution of Gi/o bound structures is dispersed compared with the Gs bound structures, indicative of less restraint of TM5-TM7 movement in response to Gi/o binding.

Currently, only four non-rhodopsin structures with β-arrestin bound (6U1N, 6PWC, 6UP7, and 6TKO) have been solved, so the distribution tendency is not clear. However, it is inferred that β-arrestin binding leads to a similar TM6 displacement with Gi/o binding rather than Gs binding[3].

As for the nanobody-bound receptor complexes, some of them cluster in the position close to GPCR-Gs complexes because they are Gs-mimic nanobodies. Nanobody-bound receptor structures can sample both the β-arrestin- and G protein-bound structure clusters. Remarkably, some nanobodies can stabilize the intermediate (nanobody 6 for succinate receptor SUCNR1) or inactive (nanobody 6 for κ-OR and nanobody 60 for β2AR) structures. These receptor structures are located at a common position for corresponding structures without nanobody.

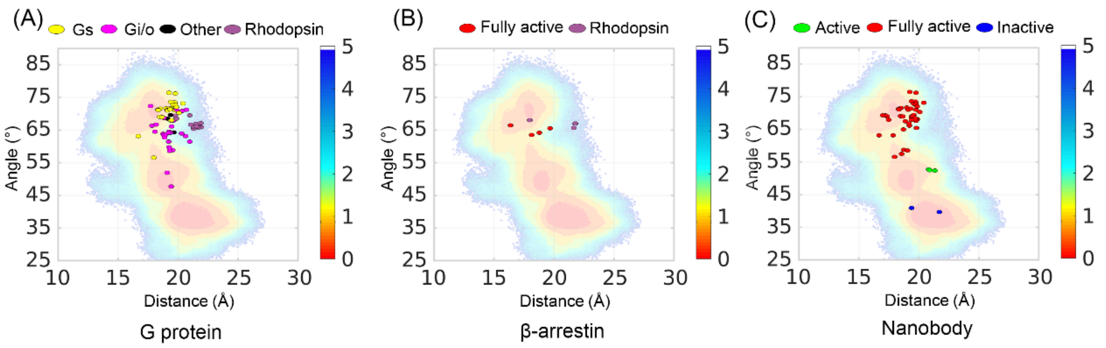

Supplementary Fig. 6: Projection of all reported G protein- (A), β-arrestin- (B) and nanobody- (C) bound GPCR structures onto the AT_1 receptor conformational landscape.

Supplementary Note 5: Validation of Markov State Model Based on the Activation Parameters

Before the construction of our MSM, we confirmed the Markovian lag time of our system via the implied timescale test. According to specified k-means cluster parameters, multiple transition probability matrixes (TPMs) were constructed corresponding to distinct lag times which represents the time interval between transitions. TPMs reflect the possibility of transition among all microstates and determine the relaxation timescales (i.e. implied timescale) through Supplementary Equation 1.

$$\tau_i = - \tau/\ln \lambda_i \qquad (1)$$

where τ represents the lag time for the computation of TPMs, λ_i is the ith eigenvalue of the TPM and τ_i means the implied timescale corresponding to the ith relaxation of the model.

For a special system, the results of eigendecomposition reflect the timescale of transition and λ_1 represents the slowest transition. As a function of the lag time τ, τ_i (especially τ_0) will be constant when the transition dynamics between microstates are Markovian[4,5]. During the test, we clustered the points into 200 microstates using the k-means algorithm and a maximum iteration number of 200. As shown in Supplementary Fig. 7A, our τ_i curves start to be flattened from a lag time than 5 ns, suggesting that a lag time longer of 5 ns confirms the Markovian properties of our activation parameters. To guarantee the accuracy of our analysis, we set 8 ns as the lag time of our system.

According to the shape of the AT_1 receptor activation pathway, we further clustered the microstates into three macrostates using the Perron Cluster Cluster Analysis (PCCA+) algorithm. Through the Chapman-Kolmogorov test shown in Supplementary Fig. 7B, the transition probability estimated by MSM is highly close to the practical transition process[6]. Thus, our MSM estimation is validated in both microstates and macrostates.

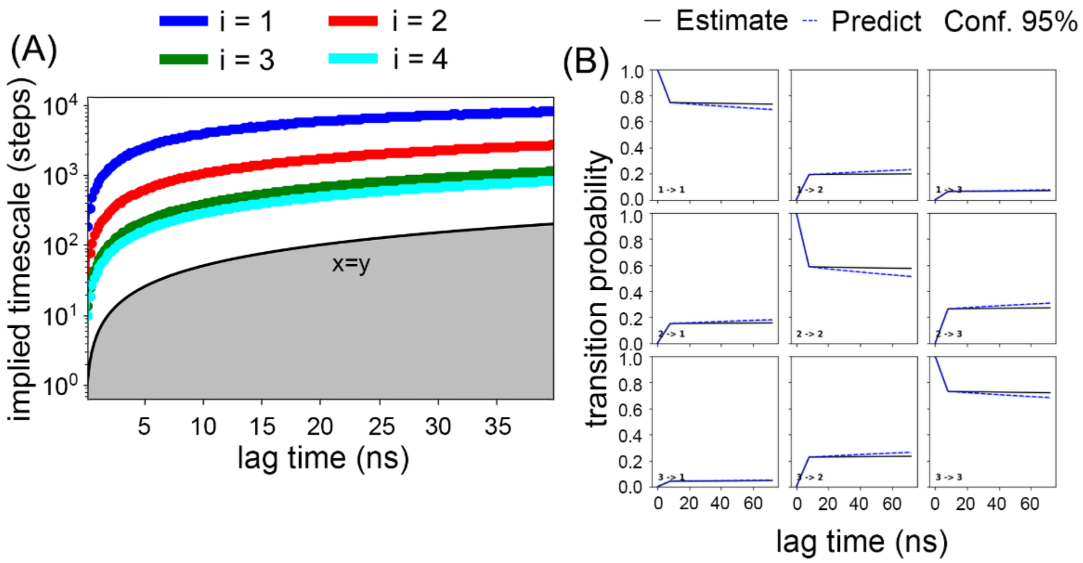

Supplementary Fig. 7: The validation of MSM accomplished by PyEMMA[7]. (A) The implied timescale test in which the timescale τ_i was shown as a function of lag time. (B) The result of the Chapman-Kolmogorov test. Estimate lines (solid) are probability calculated by MSM, while predict lines (dotted) are probability observed in trajectories.

Supplementary Note 6: Protein Levels of AT_1 Receptor and Vasopressin 2 Receptor (V_2R)

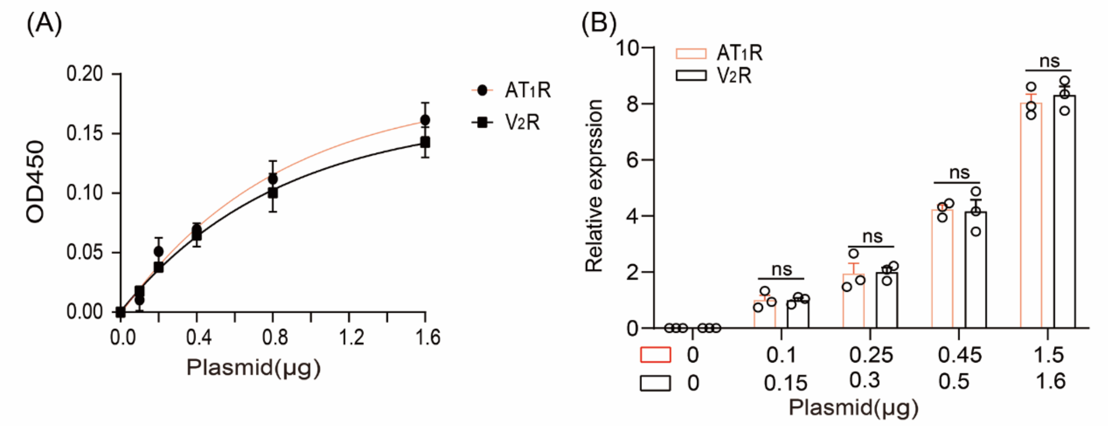

Supplementary Fig. 8: (A) Cell surface expression levels of AT_1 receptor and vasopressin 2 receptor (V_2R) measured by cell surface ELISA. Data were from three independent experiments. (B) Equal expression levels of AT_1 receptor and V_2R were achieved by adjusting the transfecting amounts in HEK293 cells. Data were from three independent experiments. n.s., no significant difference; HEK293 cells transfected with V_2R were compared with those transfected with AT_1 receptor. The bars indicate the mean ± SEM values. Statistical differences between AT_1 receptor and V_2R were analyzed using two-sided unpaired Student's t-test.

Supplementary Note 7: Position of ECL2, TM6, and H8 in Different Macrostates

We clustered inactive, active, and intermediate macrostates and observed structural variation in the ECL2, TM6, and H8 (Supplementary Fig. 9). Supplementary Fig. 9A shows the movement of ECL2 and TM6 towards the AngII pocket during activation, which finally closes the pocket. In Supplementary Fig. 9B, H8 generally moves upwards during activation in order to accommodate downstream transducers. In the inactive structure, H8 forms a large angle with TM7, which is unable to accommodate a ligand in the inactive structure. Moreover, the active macrostate has a tight space between TM1, TM7, and H8 that a pocket cannot be formed. Thus, a potential cryptic pocket may exist among TM1, TM7, and H8 in the intermediate state.

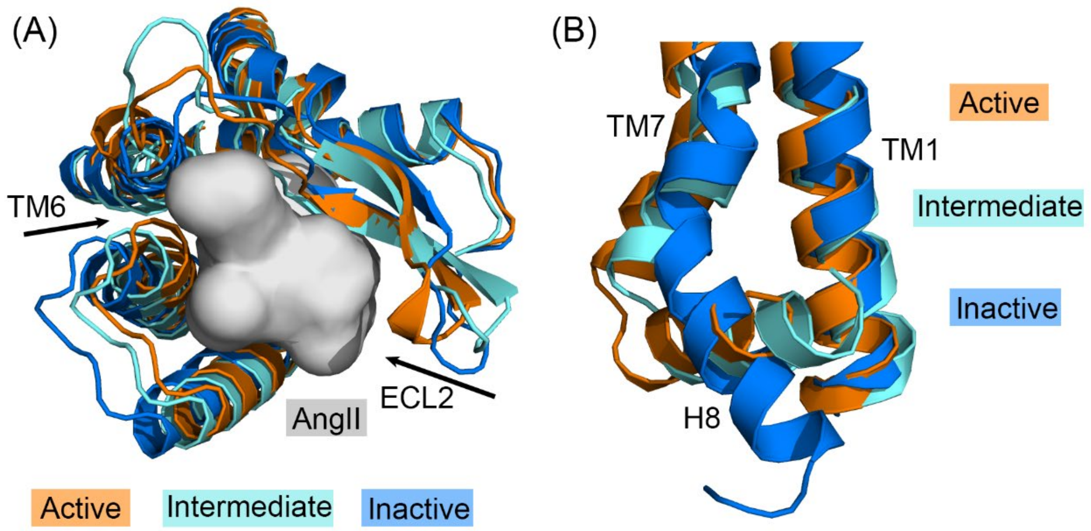

Supplementary Fig. 9: (A) Extracellular view of the macrostates. Arrows show movement during activation. N-terminal, TM1, TM2, and ECL1 are hidden for clarity. (B) The position of H8 and surrounding TMs in the macrostates.

Supplementary Note 8: Secondary Structure of Different Macrostates

Using the mdtraj package, we extracted structures corresponding to each macrostate into trajectories (see Methods). These trajectories conclude snapshots far more than single representative structures and provide more general information for different AT_1 receptor states during simulations. Thus, we applied the DSSP algorithm to reflect the flexibility of the whole structure. The output secondary structure classification of each residue in its snapshot course was shown in Supplementary Fig. 10.

Overall, the AT_1 receptor maintained its seven helical architecture, and the macrostates showed limited fluctuation when compared with themselves; this confirmed the accuracy of our classification. However, the active AT_1 receptor had a more stable ECL2 between TM4 and TM5 than both the inactive and intermediate states, thereby suggesting that the active AT_1 receptor closes the endogenous pocket better. Between TM5 and TM6, the elongation of the intracellular α-helix of TM5 positioned ICL3 in the active state. However, this part of the residue was interchanged between the α-helix, β-bend, and loop in the intermediate state. Hence, the intermediate AT_1 receptor is involved in the transition process of activation. Additionally, the boundary between TM7 and H8 was vague in the inactive state. This implies that the angle between TM7 and H8 increases and that H8 moves towards the inactive state, which is also in line with the observation of the representative structures. These key domain movements and structural variations are synergistic from the inactive to the intermediate and active states, and thus suggest a synergistic mechanism for AT_1 receptor activation.

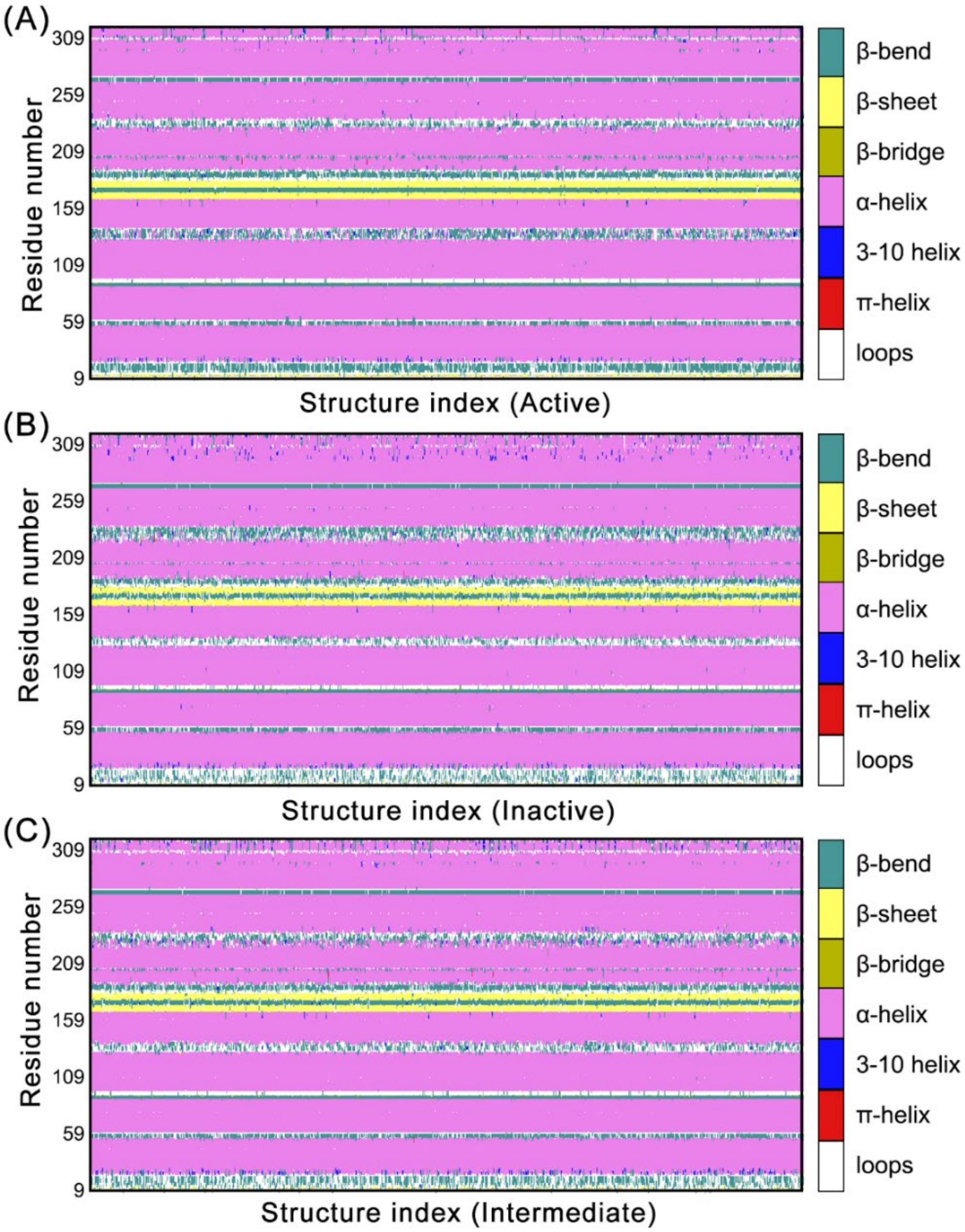

Supplementary Fig. 10: The secondary structure of representative structures around the center of every microstate in active (A), inactive (B), and intermediate (C) states, based on DSSP classification. The structure category corresponding to its colors is shown on the right. The figures were drawn by VMD.

Supplementary Note 9: Community Dynamics and Signal Transmission of Each State

Community analysis was also conducted to elucidate the signal transmission in different AT_1 receptor. During the community analysis, the community networks of distinct AT_1 receptor were determined as sets of nodes connected by weighted edges. Cα atom for each residue was defined as a node, while nodes are connected if the minimum distance between the residues were lower than 4.5 Å for at least 75% of the representative trajectories. Floyd–War-shall algorithm calculated the optimal paths among all pairs of nodes[8]. Then, we counted the pairwise optimal paths between nodes and set them as the betweenness. A community was formed if and only if a group of nodes are more intra-connected with each other than inter-connected with other nodes. With the help of the Girvan–Newman algorithm, communities were further optimized to maximize the modularity measure[9]. Of note, communities with too few (fewer than three) residues or only with connection to one other community were merged to the nearby communities. The final community distributions for AT_1 receptors are shown in Supplementary Fig. 11.

The global complexity of connection was decreased upon activation, but the key interactions among the areas were promoted in the active state. For instance, the connections between C3 (intracellular TM3 and TM5), C4 (ICL2 and around TM5 and TM6), and C5 (intracellular TM2 and TM4), which represent the interaction of the transducer pocket with other AT_1 receptor regions, were enhanced in the active state. This implies that some unnecessary interactions are quenched during activation, but the connections that transmit the activation signal to the G protein pocket are stronger. The absence of interactions between C4 and C5 in the intermediate state may reflect a transition state to form new connections. In addition, H8 was not an individual community in the active AT_1 receptor but merged itself with TM1, which reflects that it couples with other parts more during activation.

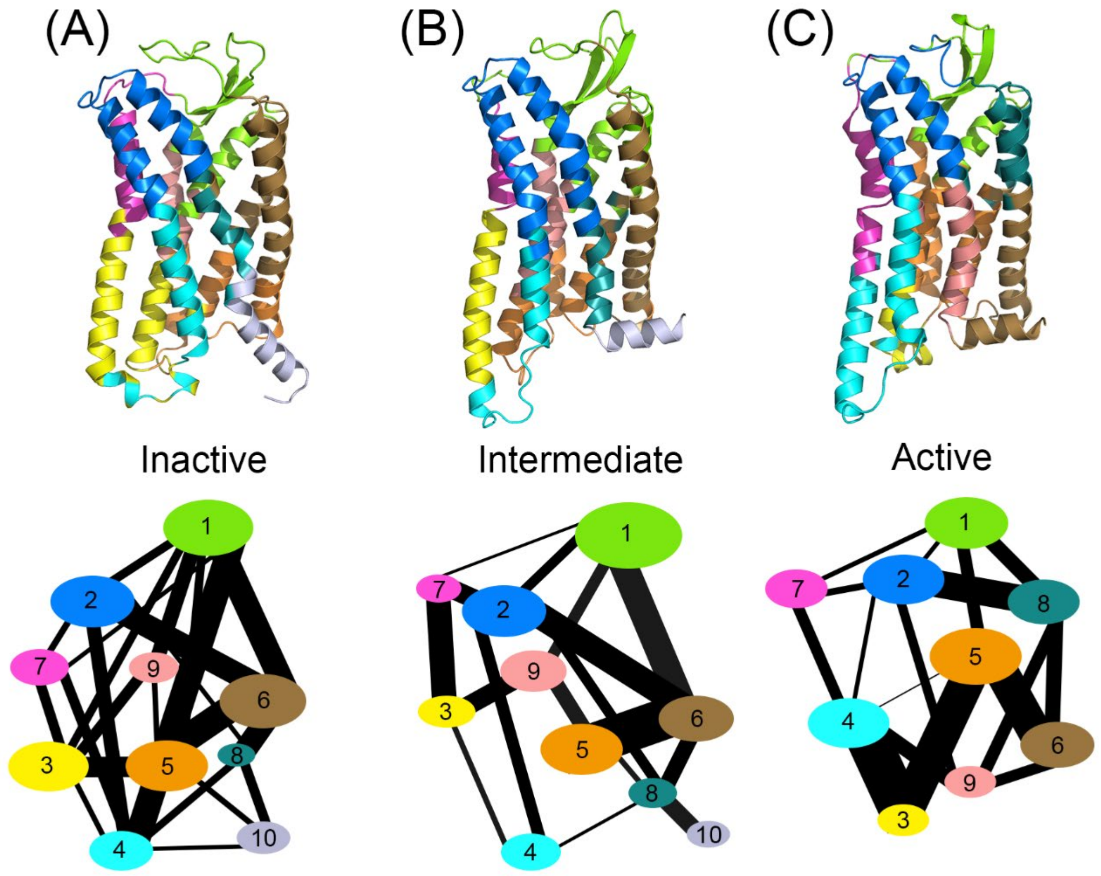

Supplementary Fig. 11: The community network of inactive (A), intermediate (B), and active (C) macrostates. Ovals represent corresponding communities with the following color scheme (C1: green, C2: blue, C3: yellow, C4: cyan, C5: orange, C6: sand, C7: magenta, C8: dark green, C9: salmon, C10: light blue, consistent with cartoon color shown on the top). The areas of ovals represent the number of residues in the community and the width of lines connecting each community is proportional to the betweenness of the communities.

Supplementary Note 10: Choice of Atoms for the Measurement of the Hydrophobic Network

To confirm the choice of atoms in the evaluation of hydrophobic network in Fig. 4E, we calculated the area among the Cβ or the Cγ atoms of $L^{3.43}$, the Cβ atom of $V^{6.41}$, and the Cβ atom of $I^{6.40}$. The comparison between the two measurement ways was shown in Supplementary Fig. 12. The tendency of area change was similar in the two curves. Thus, the choice of the atoms did not influence our description of the hydrophobic network.

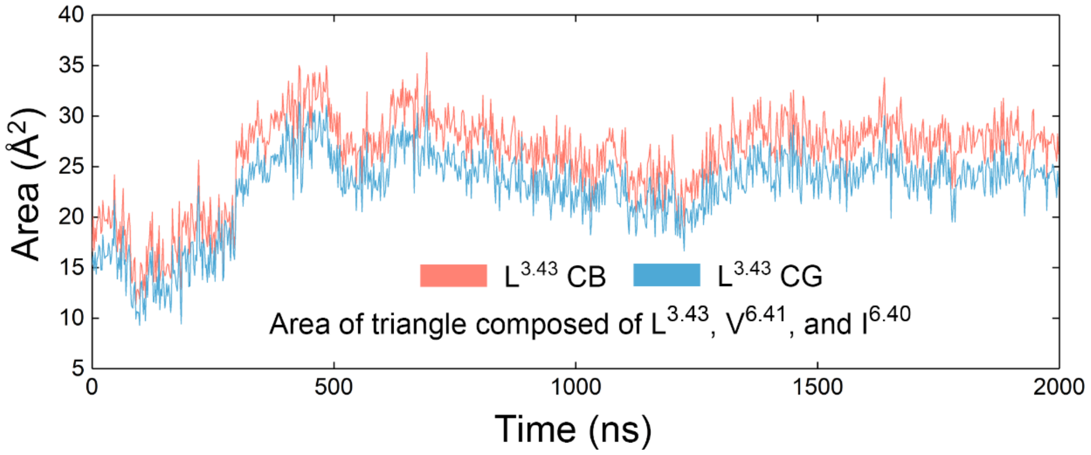

Supplementary Fig. 12: Variations of the area of the triangle composed of the Cγ (blue line) or Cβ (red line) atom of $L^{3.43}$, the Cβ atom of $V^{6.41}$, and the Cβ atom of $I^{6.40}$ in the representative trajectory. The figure was drawn by Origin.

Supplementary Note 11: Gaussian Accelerated MD Simulations for the Holo Systems

In order to further confirm the potential of the intermediate state, we designed the holo AT_1 receptor systems with an inverse agonist and an agonist bound, respectively. We first docked the inverse agonist, olmesartan, and the natural agonist, AngII, to the orthosteric pocket of the intermediate AT_1 receptor using Molecular Operating Environment. To place ligand in the pocket, the triangle matcher algorithm and London dG score were applied to obtain 60 poses. Then, we set the receptor rigid and used GBVI/WSA dG score in the refinement step. We chose the best-scored pose for system setup in the output 10 poses.

Following the system setup steps in Methods, we constructed olmesartan and AngII systems for simulation. The force field of olmesartan was generated by CHARMM General Force Field[10]. Minimization, heating, and equilibrium processes were the same as settings in Methods. Then, 2 μs conventional MD simulations were finished to further relax the system.

Gaussian accelerated MD (GaMD) is an enhanced sampling algorithm in simulation and successfully used in GPCR systems[11,12]. During GaMD, a harmonic boost potential is added to the system when the system potential $V(r)$ is lower than E_{thresh}.

$$V^*(r) = V(r), \quad V(r) \geqslant E_{thresh} \tag{2}$$

$$V^*(r) = V(r) + \Delta V(r), \quad V(r) < E_{thresh} \tag{3}$$

$$\Delta V(r) = \frac{1}{2}k(E_{thresh} - V(r))^2 \tag{4}$$

where k is the harmonic force constant. E_{thresh} and k are adjustable parameters determined by two criteria. One is the extra energy does not change the previous energy sequence of each conformation, the other is the boost potential decreases the energy distinction between conformations. Thus, the range of k and E_{thresh} is set by Supplementary Equation 5.

$$V_{max} \leqslant E_{thresh} \leqslant V_{min} + \frac{1}{k} \tag{5}$$

where V_{max} and V_{min} are the system maximum and minimum potential energies, respectively. To make Supplementary Equation 5 valid, Supplementary Equation 6 should be guaranteed.

$$k = k_0 \frac{1}{V_{max} - V_{min}} \quad (6)$$

where k_0 is determined by Supplementary Equation 7.

$$k_0 = \min\left(1.0, \frac{\sigma_0}{\sigma_V} \times \frac{V_{max} - V_{min}}{V_{max} - V_{avg}}\right) \quad (7)$$

In Supplementary Equation 7, V_{avg} and σ_V are the average and standard variation of potential energies, while σ_0 is a user-defined upper limit.

Our independent three rounds of GaMD runs started with randomized initial atomic velocities. We boosted total potential and dihedral energy in simulation. The average and standard variation of energy were calculated every 400 ps, while the upper limit was 6.0 kcal/mol. In each run, 26-ns short conventional MD simulation was firstly applied to evaluate potential energies for acceleration parameters, then a 50-ns equilibration run with the boost energy was employed. The production runs are 1 μs and the sampling timestep was 100 ps.

After simulations, we also calculated the activation parameters (distance between the Cα atoms of $L^{5.55}$ and $N^{7.46}$, the angle between the Cα atoms of $F^{6.34}$, $S^{6.47}$, and $V^{2.41}$) for each system. The corresponding free energy landscapes were shown in Supplementary Fig. 13. In the AngII binding, the TM6 angle of the AT_1 receptor largely adopted more than 55° and the tendency fitted the outward movement of TM6, which resembles to the active state. On the contrary, olmesartan binding rendered the AT_1 receptor less active in the TM6 angle and moved the free-energy landscape downwards. As for the distance index, major conformers (darkest part) of the AngII-bound AT_1 receptor are around 15-17 Å, which are close to the active conformation. However, olmesartan binding maintained the distance value in the range of 18-20 Å, consistent with the feature of the inactive conformations. Thus, inverse agonist and agonist binding shifted the intermediate state towards the inactive and active states, respectively. Together, these results suggested the reasonability of the intermediate structure captured from the transition pathway.

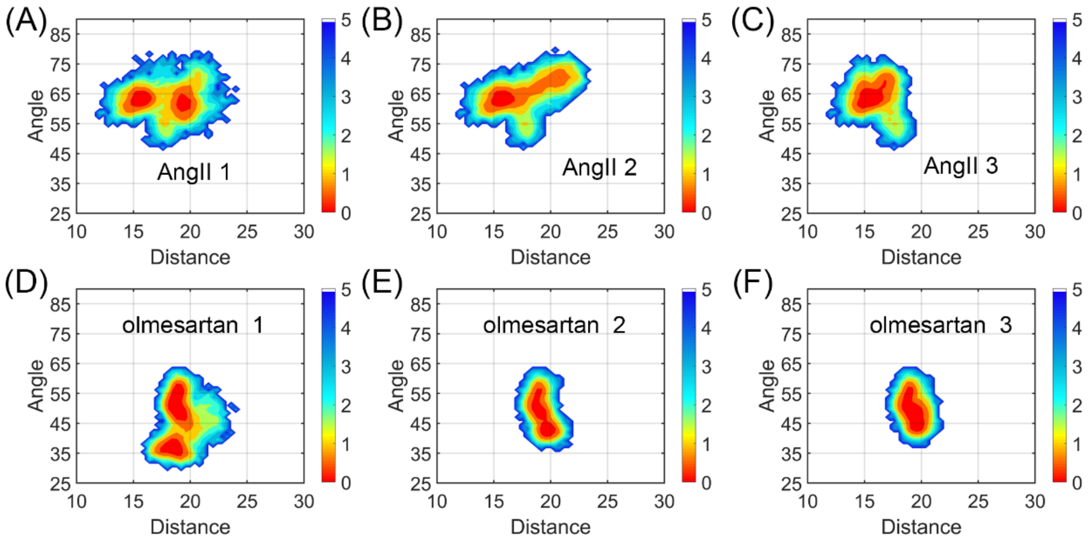

Supplementary Fig. 13: The conformational landscape of AT_1 receptor generated using the Cα atom distance between $L^{5.55}$ and $N^{7.46}$, and the angle among the Cα atoms of $F^{6.34}$, $S^{6.47}$, and $V^{2.41}$ as the order parameters along the activation pathway. (A–C) GaMD rounds 1-3 in the intermediate AT_1 receptor with AngII bound. (D–F) GaMD rounds 1-3 in the intermediate AT_1 receptor with olmesartan bound.

Supplementary Note 12: Intermediate-specific Interactions in the Other Two States

We compared the three macrostates and found several intermediate-specific micro-switches (Supplementary Fig. 14). These micro-switches exist in the intermediate state due to their unique conformation. During activation, TM5 moves towards TM7 and becomes close to TM6 in its intermediate state, in which the polar contacts among $K^{5.42}$, $H^{6.51}$, and $T^{6.55}$ form (Supplementary Fig. 14A). In addition, the TM6 outward movement generates a hydrogen bond between $I^{6.37}$ and $Y^{5.58}$ in the intermediate state (Supplementary Fig. 14B). TM6 movement also causes the formation of a hydrophobic network among $M^{6.38}$, $W^{5.62}$, and $F^{6.34}$ in the intracellular intermediate structure (Supplementary Fig. 14C). As for the H8 movement, the hydrophobic contacts among $V^{1.53}$, $V^{1.56}$, and $F^{8.50}$ only exist in the intermediate state (Supplementary Fig. 14D).

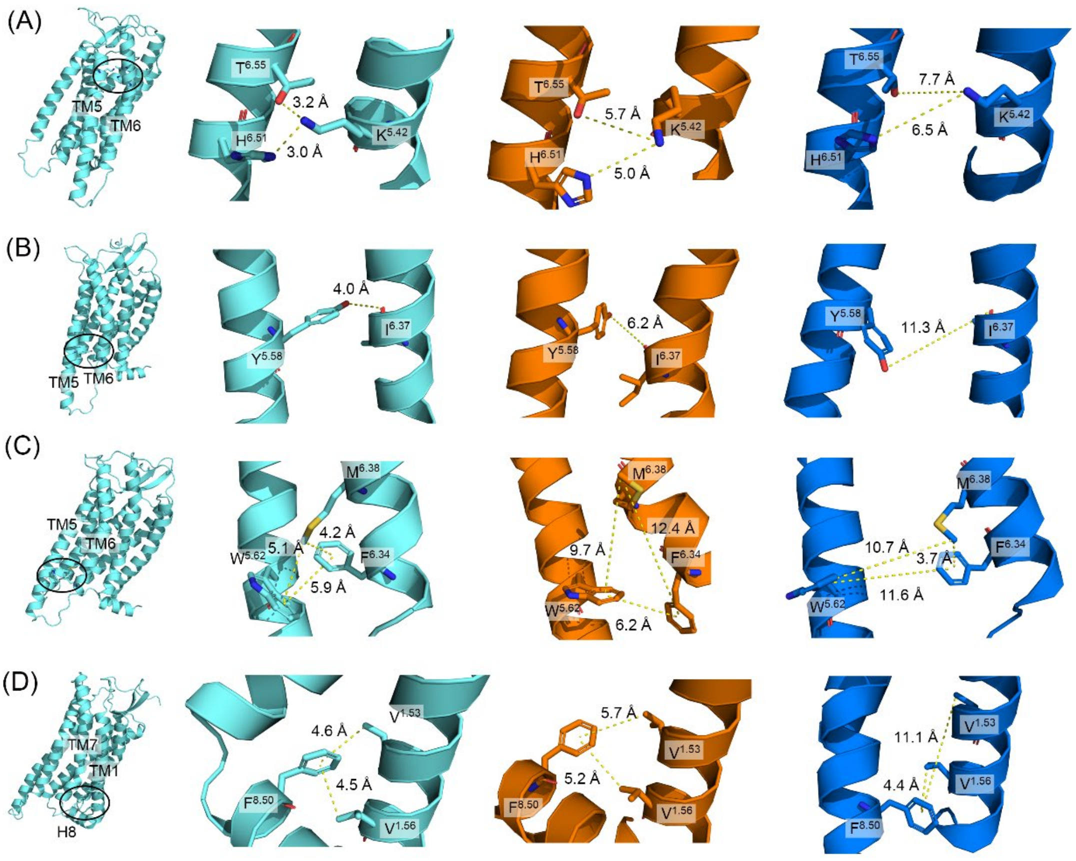

Supplementary Fig. 14: Intermediate-specific interactions (cyan) in other two active (orange) and inactive (blue) states. (A) The polar contacts among $K^{5.42}$, $H^{6.51}$, and $T^{6.55}$. (B) The hydrogen bond between $Y^{5.58}$ and $I^{6.37}$. (C) The hydrophobic network among $M^{6.38}$, $W^{5.62}$, and $F^{6.34}$. (D) The hydrophobic contacts among $V^{1.53}$, $V^{1.56}$, and $F^{8.50}$.

Supplementary Note 13: Protein Levels of WT AT_1 Receptor and Variants for the Intermediate-specific Micro-switches

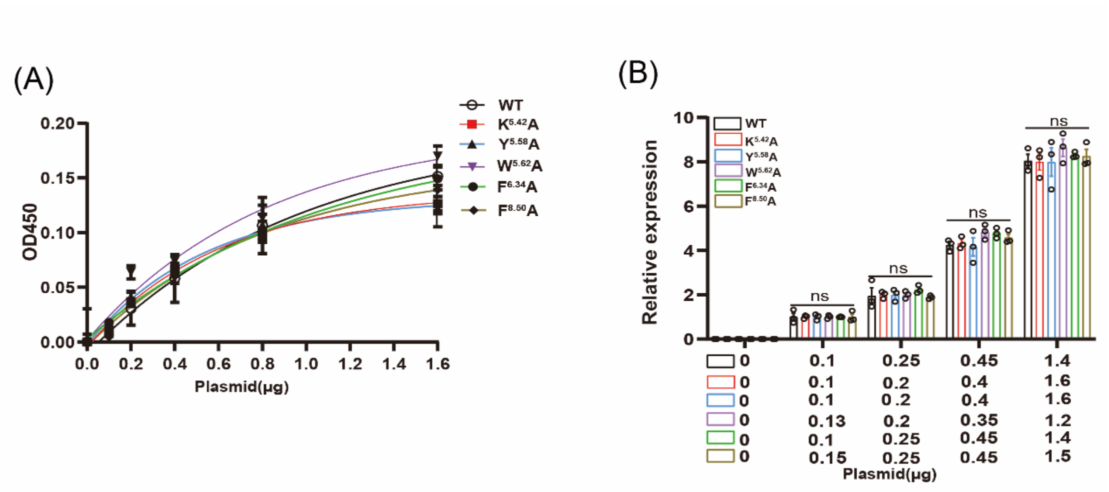

Supplementary Fig. 15: (A) Cell surface expression levels of WT AT_1 receptor and mutants measured by ELISA. Data were from three independent experiments. (B) Equal expression levels of WT AT_1 receptor and mutants were achieved by adjusting the transfecting amounts in HEK293 cells. Data were from three independent experiments. n.s., no significant difference; HEK293 cells transfected with AT_1 receptor mutants were compared with those transfected with the WT AT_1 receptor. The bars indicate the mean ± SEM values. Statistical differences between WT and mutants were analyzed using one-way ANOVA with Dunnett's post hoc test.

Supplementary Note 14: WT AT_1 Receptor and Variants for the Intermediate-specific Micro-switches

Data in Supplementary Table 1 listed the mean EC_{50} (EC_{50} ± SEM), and E_{max} (E_{max} ± SEM). Data are from three independent experiments. The E_{max} and surface expression of AT_1 receptor mutants were normalized to WT. *P < 0.05; **P < 0.01; ***P < 0.001; HEK293 cells transfect with AT_1 receptor mutants were compared with those transfected with WT AT_1 receptor. Statistical differences between WT and mutants were analyzed using one-way ANOVA with Dunnett's post hoc test (The p values for $K^{5.42}A$ are as follow. Gq-EC50: p=0.0013; Gq-Emax: p=0.0020; Gi-EC50: p<0.0001; Gi-Emax: p=0.0004; G12-EC50: p=0.0368; G12-Emax: p=0.0011. For $Y^{5.58}A$, from left to right, p<0.0001, <0.0001, <0.0001, =0.0009, 0.0040, <0.0001. For $W^{5.62}A$, from left to right, p=0.0002, 0.0572, 0.0009, 0.0593, 0.0001, <0.0001. For $F^{6.34}A$, from left to right, p<0.0001, =0.1612, 0.0007, 0.2532, 0.0227, <0.0001. For $F^{8.50}A$, from left to right, p=0.0004, 0.0059, 0.0004, 0.0209, <0.0001, <0.0001.).

Supplementary Table 1. The EC_{50} and E_{max} values of the BRET assays for Gq, Gi, and G12.

	Gq		*Gi*		*G12*	
Mutation	EC_{50} (nM)	E_{max} (%)	EC_{50} (nM)	E_{max} (%)	EC_{50} (nM)	E_{max} (%)
WT	8.87±0.49	100	10.09±0.75	100	11.32±0.69	100
$K^{5.42}A$	20.92±1.40**	40.33±2.67**	222.70±11.42***	43.33±1.20***	14.18±0.61*	70.00±1.00**
$Y^{5.58}A$	50.45±0.50***	53.33±0.33***	105.00±0.75***	54.67±1.33***	17.07±0.67**	29.67±0.33***
$W^{5.62}A$	19.87±0.71***	96.00±1.00	16.77±0.14***	116.00±3.00	24.39±0.54***	33.67±0.33***
$F^{6.34}A$	32.92±1.30***	88.00±5.51	21.18±0.90***	104.30±2.72	14.79±0.67*	46.67±0.33***
$F^{8.50}A$	16.08±0.44***	85.00±1.16**	20.18±0.50***	82.00±2.65*	156.50±5.40***	33.67±0.33***

Supplementary Note 15: TICA Analysis of Trajectories

To build an MSM based on tICA, we firstly aligned structures on the first one to elucidate the influence of translation and rotation, then we featurized the Cartesian coordinates in trajectories to decrease the dimension. After the alignment finished by CPPTRAJ, the "add_backbone_torsions" function provided by PyEMMA was used to extract all backbone phi/psi angles during simulation, reflecting the global movement of the membrane-embedded AT_1 receptor[7,13]. Next, considering the great ability of tICA to describe slow dynamics in simulation, the "coordinates_tica" method was applied for dimensionality reduction to 2-dimension[14]. Referred to the implied timescale test for activation parameter, Supplementary Equation 1 was applied for the Markovian property test of tICA landscape with 200 cluster centers and 200 maximum k-means iterations. As shown in Supplementary Fig. 6A, the τ_i curves of the AT_1 receptor become independent of lag time from 5 ns. Consequently, a lag time longer than 5 ns confirms tICA landscape Markovian and we also set 8 ns as the lag time for the creation of MSM using the "msm.estimate_markov_model" function.

After plotting the Markovian tIC1 and tIC2 on Supplementary Fig. 17A to show the global movement ensemble, we also employed the PCCA+ algorithm to the systems and clustered it into eight macrostates. The distribution of them is shown in Supplementary Fig. 17B. Similarly, the Chapman-Kolmogorov test (Supplementary Fig. 16B) proved that the 8-macrostate model matches the practical transformation that happened in simulation. Next, representative trajectories for macrostates were extracted by mdtraj using snapshots close to k-means centers. According to S_{ij} calculated by Eq. (5), the structure with the most S_{ij} on each trajectory was identified as the representative structure, which was projected onto Fig. 5A according to its activation parameter. With the help of the MFPT algorithm, the transition time among macrostates was determined by the "msm.estimate_markov_model.mfpt" method.

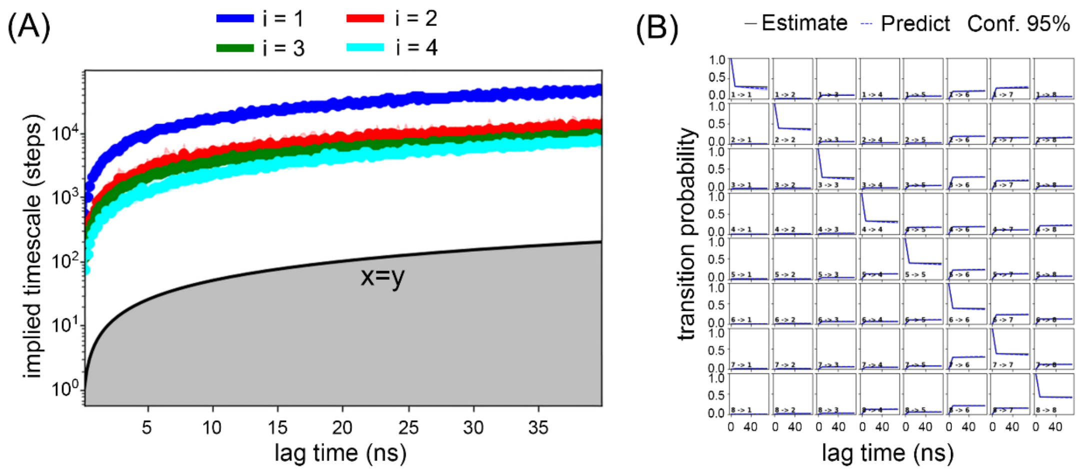

Supplementary Fig. 16: The validation of an MSM constructed by tICA. (A) The result of the implied timescale test. Different timescales τ_1, τ_2, τ_3, and τ_4 were represented as blue, red, green, and cyan lines changing with lag time. (B) The result of the 8-state Chapman-Kolmogorov test. MSM estimates the solid probability lines, while dotted predict lines are transition probability observed in simulations.

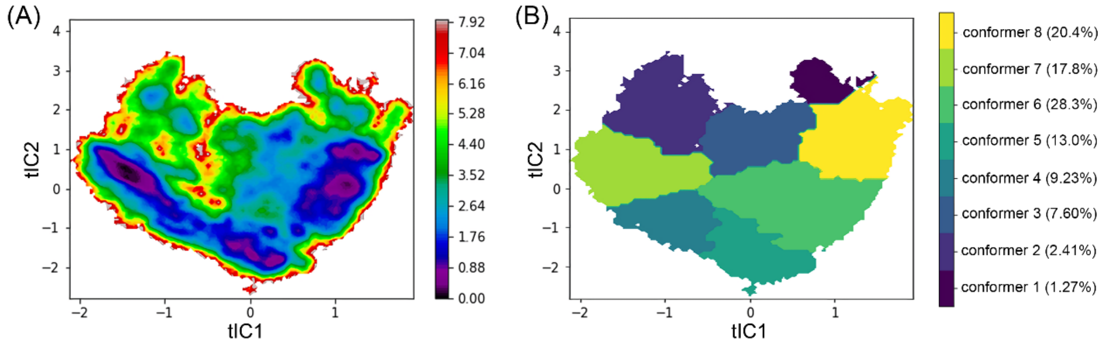

Supplementary Fig. 17: (A) 2-D free energy landscape of AT_1 receptor simulation based on the first and second tICA components (tIC1 and tIC2). The unit of free energy is kcal/mol. (B) The distribution of eight macrostates on the tICA landscape. Corresponding color and proportion are shown on the right.

Supplementary Note 16: Construction of AT_1 Receptor-Transducer System

With respect to a recent publication[15], the structure of M1 muscarinic acetylcholine receptor (M1AChR) bound to the G protein (PDB ID: 6OIJ) was used to construct Gq-bound AT_1 receptor models[16]. Similarly, the β-arrestin-bound AT_1 receptor model was constructed based on the rhodopsin-arrestin structure (PDB ID: 4ZWJ)[17]. To highlight the significant area, we only modeled the α5 helix of Gq and the finger loop of β-arrestin 1 because they are the only regions inserting into the intracellular side of GPCRs.

During the modeling, we first selected all residues on the GPCRs with the transducer bound. Then, maintaining the relative position of GPCR and downstream proteins, we aligned the object GPCR to the representative structure of the AT_1 receptor, referred to all Cαs in the AT_1 receptor. The alignment was finally refined in 5 cycles of Executive RMS provided by PyMOL.

As shown in Supplementary Fig. 18, the macrostates 6 and 7 correspond to the Gq-bound and β-arrestin 2-bound conformations, respectively. In macrostate 6, the sidechain of $R^{3.50}$ of AT_1 receptor was horizontal and accommodated the α5 helix of the Gq protein. In sharp contrast, $R^{3.50}$ in macrostate 7 tilted towards the intracellular side and clashed with Gq. As a result, macrostate 7 was resistant to binding to Gq, consistent with the definition of the β-arrestin 2-bound active conformation[15]. Thus, we showed that both the Gq-bound and β-arrestin 2-bound active conformations exist in the apo AT_1 receptor.

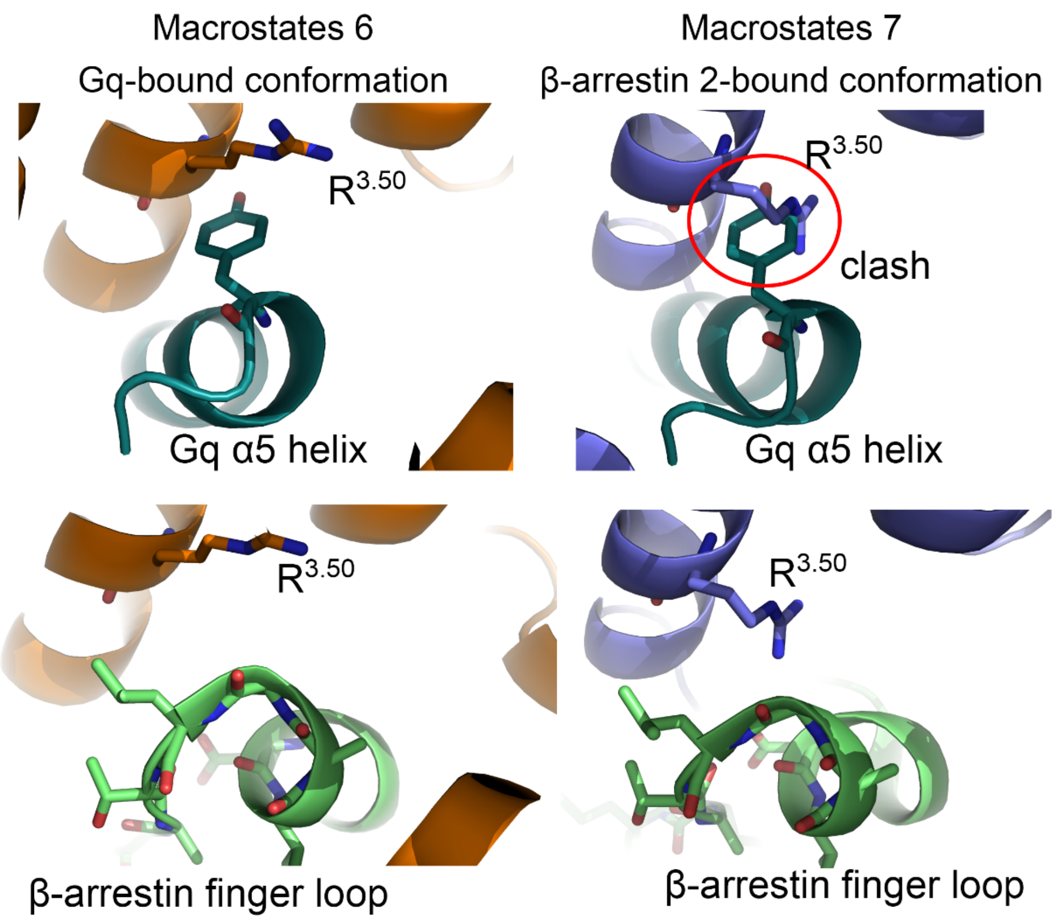

Supplementary Fig. 18: The zoom-in view of the transducer pocket of AT_1 receptor-Gq and β-arrestin 2 structure models. Macrostate 6 is depicted by orange cartoon and macrostate 7 is shown as purple cartoon. Gq and β-arrestin 2 are colored in dark and light green, respectively. The clash between $R^{3.50}$ and Gq in the β-arrestin 2-bound conformation is shown in a red circle.

Supplementary Note 17: AngII-induced Activation on WT AT_1 Receptor and Variants for Different tICA Macrostates

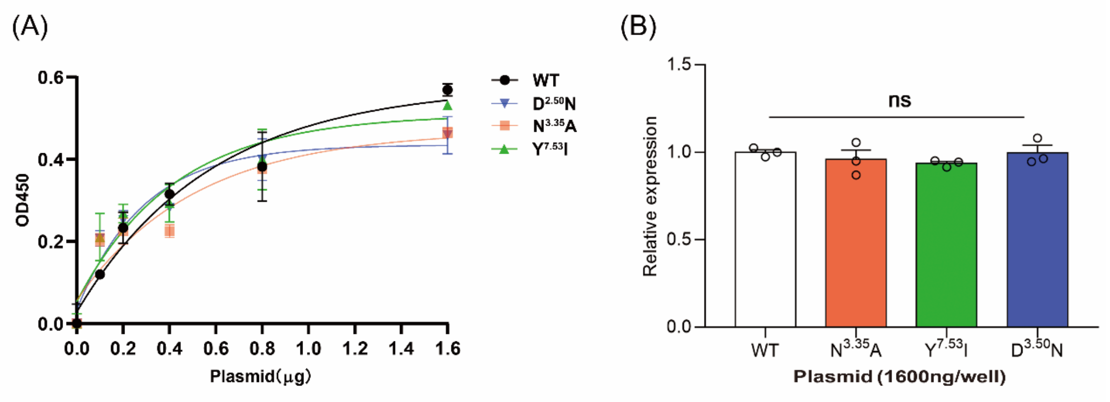

Supplementary Fig. 19: (A) Cell surface expression levels of WT AT_1 receptor and mutants measured by ELISA. Data were from three independent experiments. (B) Equal expression levels of WT AT_1 receptor and mutants were achieved by controlling the transfecting amounts in HEK293 cells. Data were from three independent experiments. n.s., no significant difference; HEK293 cells transfected with AT_1 receptor mutants were compared with those transfected with WT AT_1 receptor. The bars indicate the mean ± SEM values. Statistical differences between WT and mutants were analyzed using one-way ANOVA with Dunnett's post hoc test.

Data in Supplementary Table 2 represent mean EC_{50} (EC_{50} ± SEM) and E_{max} (E_{max} ± SEM) for Gq, β-arrestin 2, Gi, and G12 pathways. Data are from three independent experiments. The E_{max} and surface expression of AT_1 receptor mutants were normalized to WT. *P < 0.05; **P < 0.01; ***P < 0.001; HEK293 cells transfect with AT_1 receptor mutants compared with those transfected with WT AT_1 receptor. Statistical differences between WT and mutants were analyzed using one-way ANOVA with Dunnett's post hoc test (The p values for $D^{2.50}N$ are as follow. Gq-EC50: p=0.0006; Gq-Emax: p=0.0429; β-arrestin2-EC50: p=0.0486; β-arrestin2-Emax: p=0.0317; Gi-EC50: p<0.0001; Gi-Emax: p=0.0014; G12-EC50: p=0.0108; G12-Emax: p=0.0012. For $N^{3.35}A$, from left to right, p=0.0014, 0.0041, 0.0049, 0.0008. 0.0077, 0.0008, <0.0001,

<0.0001. For $Y^{7.53}I$, from left to right, p=0.9220, 0.0006, 0.0356, 0.0785, <0.0001, 0.0013, <0.0001, 0.0057).

Supplementary Table 2. The EC_{50} and E_{max} values of the BRET assay for Gq, β-arrestin 2, Gi, and G12

	Gq BRET		*β-arrestin 2*		*Gi BRET*		*G12 BRET*	
Mutation	EC_{50} (nM)	E_{max} (%)	EC_{50} (nM)	E_{max} (%)	EC_{50} (nM)	E_{max} (%)	EC_{50} (nM)	E_{max} (%)
WT	7.13±0.54	100	8.11±0.37	100	10.06±0.48	100	10.06±0.05	100
$D^{2.50}N$	15.02±0.59***	83.33±3.76*	10.24±0.66*	76.67±4.26*	131.90±4.77***	73.00±1.00**	10.93±0.19*	83.00±0.58**
$N^{3.35}A$	2.61±0.17**	140.70±1.76**	39.58±5.58**	23.67±2.19***	16.75±1.26**	135.00±1.00***	61.62±1.18***	53.67±0.33***
$Y^{7.53}I$	7.03±1.63	72.33±0.67***	25.66±5.62*	85.33±4.37	586.50±35.91***	66.67±1.20**	17.73±0.03***	88.33±0.88**

Data in Supplementary Table 3 show the transduction coefficients of WT AT_1 receptor or mutants for activating different downstream pathways in response to AngII stimulation. Concentration-response data from were analyzed by nonlinear regression using the operational model equation in GraphPad Prism with WT AT_1 receptor as the reference receptor to calculate the transduction coefficient [$\log(\tau/K_A)$]. $\Delta\log(\tau/K_A)$ was calculated by subtracting the $\log(\tau/K_A)$ value of WT AT_1 receptor in each pathway.

Supplementary Table 3. The log(τ/K_A) and Δlog(τ/K_A) values of the BRET assay for biased signaling in Gq, β-arrestin 2, Gi, and G12

	log(τ/K_A)				Δlog(τ/K_A)			
Mutation	Gq	Gi	G12	β-arrestin 2	Gq	Gi	G12	β-arrestin 2
WT	6.25±0.25	6.36±0.02	8.17±0.01	7.89±0.02	0.00±0.35	0.00±0.03	0.00±0.01	0.00±0.03
$D^{2.50}N$	4.93±0.35	4.86±0.00	7.41±0.01	7.75±0.05	-1.69±0.43	-1.50±0.02	-0.76±0.02	-0.15±0.06
$N^{3.35}A$	8.47±0.13	7.05±0.01	5.28±0.02	4.70±0.16	1.85±0.28	0.69±0.03	-2.89±0.02	-3.20±0.16
$Y^{7.53}I$	6.12±0.09	3.62±0.01	7.82±0.02	7.32±0.15	-0.50±0.26	-2.74±0.02	-0.35±0.02	-0.58±0.15

Supplementary Note 18: Pocket Location in the Three Macrostates

Using Fpocket, we identified the potential pockets of inactive, active, and intermediate macrostates[18]. To determine the pockets only belonging to the intermediate state, we compared the result of prediction among the three states. Supplementary Table 4 depicts the detailed position of each pocket in the intermediate state. In Supplementary Fig. 20, the location of every overlapping pocket in the intermediate state is shown. According to the condition shown in Supplementary Fig. 20 and Supplementary Table 4, P6 and P9 are defined as the pockets only existing in the intermediate state.

Supplementary Table 4. Summary of pocket sites in the intermediate state

Pocket name	*Pocket Position*	*Pocket overlap with other AT_1 receptors*	*Pocket overlap with other class A GPCRs with allosteric modulators*
P1	Inside the whole AT_1 receptor	Active and inactive	All class A GPCRs
P2	Between TM7 and N-terminal, extracellular	Active	M2R
P3	Between the middle of TM5 and TM6, the membrane side	Active	None
P4	Between the top of TM1 and TM2, the membrane side	Active	$P2Y_1R$
P5	Between the bottom of TM5 and TM6, the membrane side	Active and inactive	None
P6	Between TM7, H8 and TM1	None	None
P7	Between TM3, TM5, and TM6, intracellular	Active	None
P8	Between ICL2, TM2, TM3, and TM4, intracellular	Active and inactive	AP8
P9	Between TM1, TM2, and TM7, intracellular	None	CCR2, CCR9, and β2AR

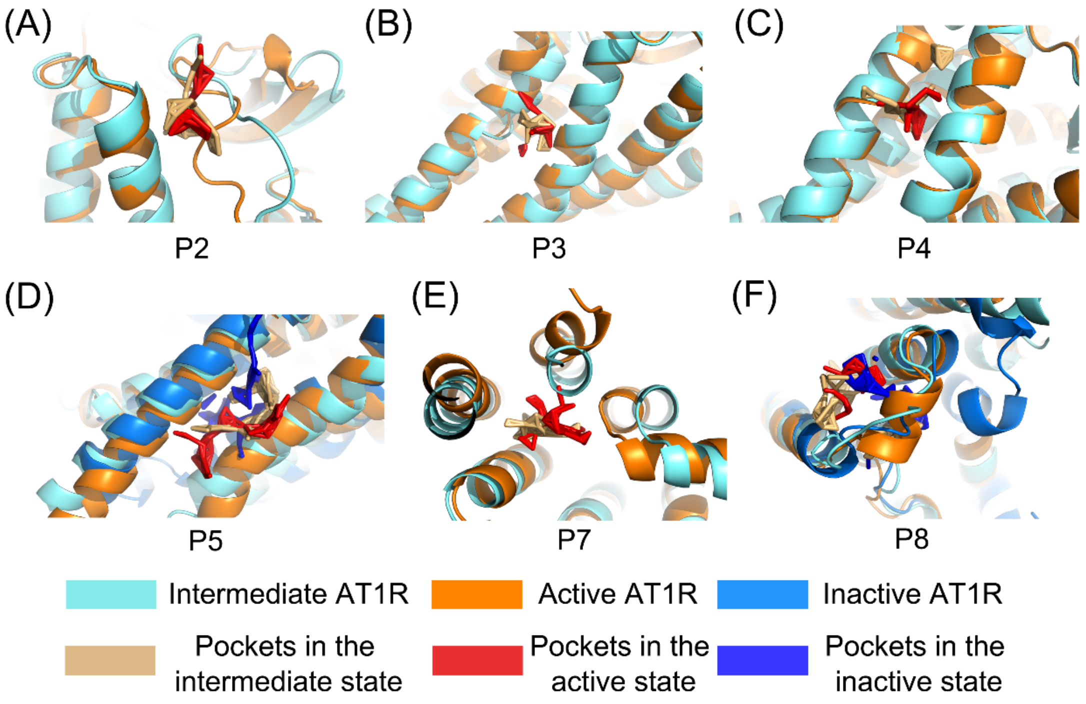

Supplementary Fig. 20: The overlapping pockets predicted by Fpocket in the active, inactive, and intermediate states. (A), (B), (C), (D), (E), (F) show P2, P3, P4, P5, P7, P8 pockets, respectively. Cyan, orange, and blue cartoons depict intermediate, active, and inactive AT_1 receptors, respectively, while their overlapping pockets are in light sand, red, and purple and represented by sticks.

Supplementary Note 19: Molecular Docking towards P6 pocket

We also conducted molecular screening on P6 using the allosteric GPCR sublibrary of Enamine to confirm the pocket and provide insights for mutations. During the screening, 14,535 compounds were firstly prepared in LigPrep model at pH 7.0, using OPLS3 force field. Then, docking in standard precision (SP) was applied for all compounds and 1,000 compounds with top-score were picked. At last, extra precision (XP) docking was employed to the 1,000 compounds and wrote the final output. Supplementary Fig. 21 shows the binding pose of compound Z367028310 with the best docking score (-5.59). The docking procedure was performed by glide in Maestro, Schrödinger suites. As seen, hydrophobic interactions dominate the binding and the binding pose highly overlaps with P6.

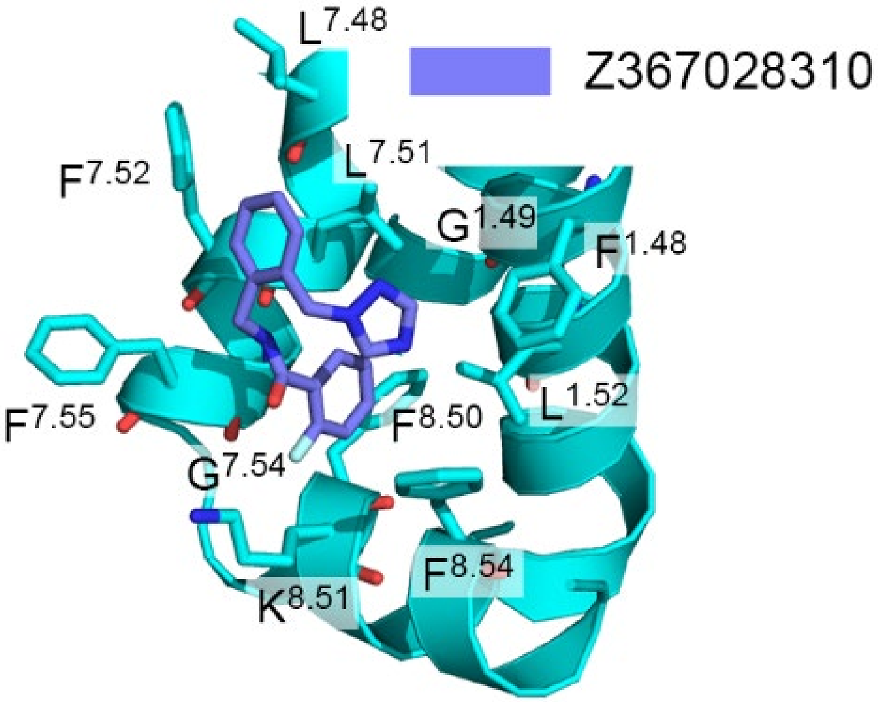

Supplementary Fig. 21: The binding pose of Z367028310 on P6 with key interaction residues shown as sticks. Z367028310 is shown in purple sticks.

Supplementary Note 20: Clustered Mutation Experiment of P6 in the AT_1 Receptor

In order to confirm the existence of P6, we first designed 3 direct mutation clusters around P6 and tested the corresponding influence on Gq and β-arrestin 2 signal, respectively. As shown in Supplementary Fig. 22, whole-cell enzyme-linked immunosorbent assay (ELISA) assay indicates that the mutant AT_1 receptors are expressed to a similar degree, compared with the WT AT_1 receptor. Thus, the variation in the downstream signaling was not caused by the change in AT_1 receptor expression level.

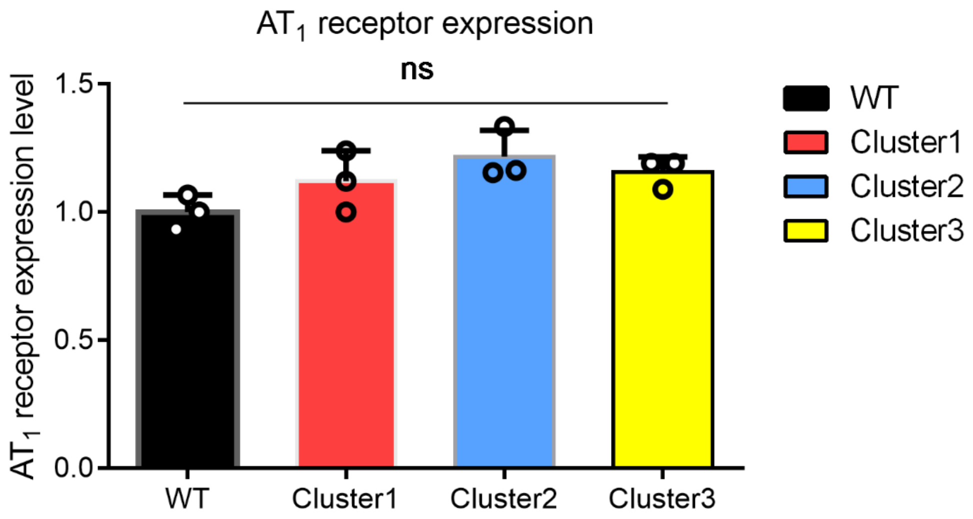

Supplementary Fig. 22: Cell surface expression levels of AT_1 receptor upon mutation cluster 1 (red), 2 (blue), and 3 (yellow). Standard deviations are shown as black lines. The expression levels of the cluster mutants were normalized to that of the WT AT_1 receptor (black). Data were from three independent experiments. ns, no significant difference; HEK293 cells transfect with AT1R cluster mutants were compared with those transfected with WT AT1R. The bars indicate the mean ± SEM values. Statistical differences were analyzed using one-way ANOVA with Dunnett's post hoc test.

Supplementary Table 5. The EC_{50} and E_{max} values of the BRET assays for Gq and β-arrestin 2 upon cluster mutations

	Gq BRET		*β-arrestin 2 BRET*	
Mutation	EC_{50} (nM)	E_{max} (%)	EC_{50} (nM)	E_{max} (%)
WT	6.19±0.32	100	2.14±0.33	100
Cluster 1	6.12±0.58	64.00±1.73**	2.43±0.13	77.33±1.20**
Cluster 2	ND	ND	ND	ND
Cluster 3	ND	ND	ND	ND

Data in Supplementary Table 5 represent mean EC_{50} (EC_{50} ± SEM) and E_{max} (E_{max} ± SEM).

**P < 0.01; n.s., no significant difference; ND, not detectable; HEK293 cells transfected with AT_1 receptor cluster mutants were compared with those transfected with WT AT_1 receptor. The bars indicate the mean ± SEM values. Statistical differences between WT and cluster mutants were analyzed using one-way ANOVA with Dunnett's post hoc test (The p values for cluster 1 are as follow. Gq-EC50: p=0.9223; Gq-Emax: p=0.0023; β-arrestin 2-EC50: p=0.4503; β-arrestin 2-Emax: p=0.0028).

Supplementary Note 21: AlloSigMA provides guidelines for the design of P6 mutations

AlloSigMA uses the Structure-Based Statistical Mechanical Model of Allostery (SBSMMA) to measure allosteric free energy. It provides the change of allosteric free energy for a specific site (such as G protein pocket) upon ligand binding, stable/bulky mutation, or unstable/tiny mutations happened on the potential allosteric site. Positive allosteric free energy reflects the increase of dynamics on the specific site or vice versa. The allosteric free energy values are shown in Supplementary Table 6.

Supplementary Table 6. AlloSigMA predicts the influence, caused by P6 ligand or mutations, on the G protein pocket. The unit is kcal/mol.

Residue number	*ΔG for G protein pocket on ligand binding*	*ΔG for G protein pocket on stable mutations*	*ΔG for G protein pocket on unstable mutations*
$F^{1.48}$	17.08	10.155	-6.925
$G^{1.49}$	8.039	7.040	-0.999
$N^{1.50}$	5.816	4.922	-0.894
$L^{1.52}$	4.077	4.718	0.641
$V^{1.53}$	3.593	3.303	-0.290
$N^{7.49}$	11.97	5.119	-6.851
$P^{7.50}$	3.500	3.481	0.020
$Y^{7.53}$	-11.04	0.426	-11.468
$G^{7.54}$	0.011	0.626	0.615
$F^{7.55}$	-12.86	-11.647	1.220
$F^{8.50}$	-2.475	3.725	6.470
$K^{8.51}$	1.916	4.917	3.000
$F^{8.54}$	0.051	5.540	5.489

As both over-fluctuation and a stable intermediate state prohibit the binding of a transducer, the larger absolute value of allosteric free energy infers corresponding mutations inhibit downstream signals. We set 5.5 kcal/mol as a significant threshold. Ligand binding mainly influences G protein pocket by TM1 ($F^{1.48}$, $G^{1.49}$, and $N^{1.50}$) and TM7 ($N^{7.49}$, $Y^{7.53}$, and $F^{7.55}$), while mutations show their obvious impact evenly on TM1 ($F^{1.48}$ and $G^{1.49}$), TM7 ($N^{7.49}$, $Y^{7.53}$, and $F^{7.55}$) and H8 ($F^{8.50}$, and $F^{8.54}$). In total, large amount of significant data in Supplementary Table 6 confirm the allosteric signal pathway between P6 and the G protein pocket. AlloSigMA also indicates that bulky mutations ($G^{1.49}$L, $F^{7.55}$W, and $F^{8.54}$W) and tiny mutations ($F^{1.48}$A, $N^{7.49}$A, $Y^{7.53}$A, and $F^{8.50}$A) can influence the dynamics of the G protein pocket. These mutations are adopted in our experiment design.

In SBSMMA, the allosteric free energy G is calculated by solving the statistical mechanical problem for protein conformation ensemble in the original state (0), ligand-bound state (B), or mutated state (M). The relations are shown in Supplementary Equation 8.

$$\Delta G_i^{(B)} = \frac{1}{2} k_B T \sum_\mu ln \frac{\varepsilon_{\mu,i}^{(B)}}{\varepsilon_{\mu,i}^{(0)}}, \Delta G_i^{(M)} = \frac{1}{2} k_B T \sum_\mu \ln \frac{\varepsilon_{\mu,i}^{(M)}}{\varepsilon_{\mu,i}^{(0)}}, \quad (8)$$

where i reflects the residue index. ε is associated with the normal modes $e_\mu^{(P)}$ and (P) indicates the state of a protein. As components of the allosteric potential U (Supplementary Equation 9), the energy values can be calculated by integrating all Cα displacements identified by σ.

$$U_i^{(P)}(\sigma) = \frac{1}{2} \sum_\mu \varepsilon_\mu^{(P)}, {}_i\sigma_\mu^2 \quad (9)$$

where σ represents a vector of Gaussian variables with variance $\frac{1}{\varepsilon_{\mu,i}^{(P)}}$, each of which is related to a corresponding normal mode. The detailed calculation was described in the original references[19,20].

G protein pocket is composed of the lower part of TM3, 5 and 6 based on the intermediate structure. The residues are $R^{3.50}$-$H^{3.56}$, $W^{5.62}$-$Q^{229\text{-}ICL3}$, and $I^{6.33}$-$I^{6.40}$. Because ICLs highly fluctuate in solution, they are not suitable for the evaluation of

dynamics parameters such as allosteric free energy.

Supplementary Note 22: AngII-induced Activation on Wild Type AT_1 Receptor and Variants for P6

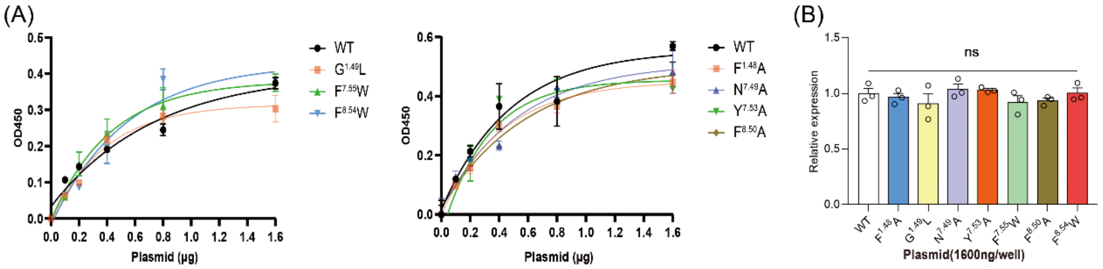

Supplementary Fig. 23: (A) Cell surface expression levels of WT AT_1 receptor and mutants measured by ELISA. Data were from three independent experiments. (B) Equal expression levels of WT AT_1 receptor and mutants were achieved by controlling the transfecting amounts in HEK293 cells. Data were from three independent experiments. n.s., no significant difference; HEK293 cells transfected with AT_1 receptor mutants were compared with those transfected with WT AT_1 receptor. The bars indicate the mean ± SEM values. Statistical differences between WT and mutants were analyzed using one-way ANOVA with Dunnett's post hoc test.

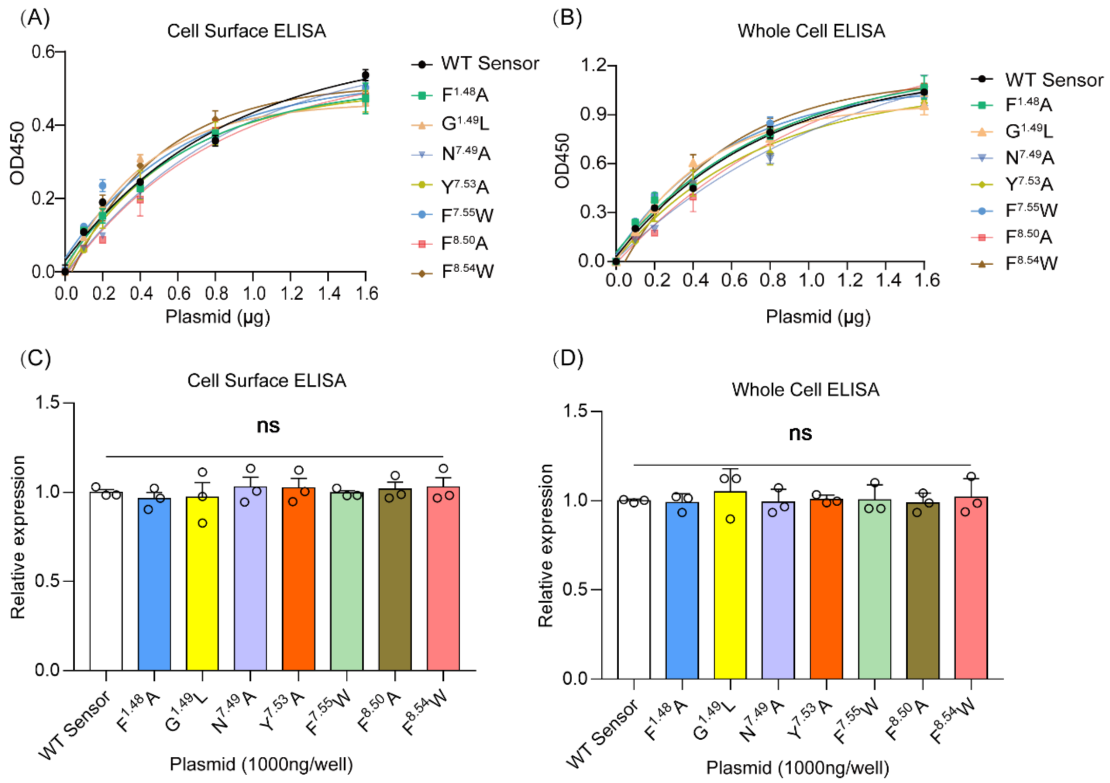

Supplementary Fig. 24: (A-B) Cell surface and total expression levels of AT_1R conformational sensor and sensor-based mutants measured by Cell surface ELISA (A) and whole cell ELISA (B). Data were from three independent experiments. (C-D) Cell surface ELISA (C) and Whole cell ELISA (D) showing equal expression levels of AT_1R conformational sensor and sensor-based mutants. Data were from three independent experiments. n.s., no significant difference; HEK293 cells transfected with AT_1R conformational sensor-based mutants were compared with those transfected with the conformational sensor. The bars indicate the mean ± SEM values. Statistical differences between WT and mutants were analyzed using one-way ANOVA with Dunnett's post hoc test.

Supplementary Table 7: The EC_{50} and E_{max} values of the BRET assays for Gq and β-arrestin 2 and the FlAsH-BRET assays using the AT_1 receptor conformational sensor.

	Gq BRET		*β-arrestin 2 BRET*		*Gq FlAsH-BRET using sensor*	
Mutation	EC_{50} (nM)	E_{max} (%)	EC_{50} (nM)	E_{max} (%)	EC_{50} (nM)	E_{max} (%)
WT	8.012±0.24	100	7.87±0.22	100	36.38±0.50	100
$G^{1.49}L$	17.51±0.81***	84.67±7.54	136.70±5.31***	16.00±1.16***	62.63±1.00***	88.33±1.76*
$F^{7.55}W$	18.45±1.14***	96.67±4.84	335.50±5.50***	18.67±0.33***	84.19±1.23***	93.00±1.73
$F^{8.54}W$	13.43±1.23***	102.00±12.06	26.28±0.22***	71.33±2.60**	78.30±0.48***	95.33±1.20
$F^{1.48}A$	15.63±0.37***	53.67±3.48**	34.02±0.91***	45.33±2.40**	111.20±3.47***	45.67±0.33***
$N^{7.49}A$	27.46±1.05***	81.67±3.28*	6.15±1.00	75.33±2.33**	135.00±0.94***	58.67±0.33***
$Y^{7.53}A$	23.51±1.49***	78.00±4.36*	44.65±0.36***	22.00±0.58***	126.90±6.33***	50.67±0.67***
$F^{8.50}A$	15.43±0.32***	81.00±3.61*	33.47±0.55***	46.67±1.76**	105.70±0.58***	56.67±0.33***

Data in Supplementary Table 7 represent mean EC_{50} (EC_{50} ± SEM) and E_{max} (E_{max} ± SEM). Data are from three independent experiments. *P < 0.05; **P < 0.01; ***P < 0.001; HEK293 cells transfect with AT_1 receptor mutants or AT_1 receptor Gq-sensor-based mutants were compared with those transfected with WT AT_1 receptor or AT_1 receptor Gq sensor. Statistical differences between WT and mutants were analyzed using one-way ANOVA with Dunnett's post hoc test (The p values for $G^{1.49}L$ are as follow. Gq-EC50: p<0.0001; Gq-Emax: p=0.1788; β-arrestin2-EC50: p<0.0001; β-arrestin2-Emax: p=0.0002; FlAsH-EC50: p<0.0001; FlAsH-Emax: p=0.0221. For $F^{7.55}W$, from left to right, p<0.0001, 0.5623, <0.0001, <0.0001, <0.0001, 0.0561. For $F^{8.54}W$, from left to right, p=0.0005, 0.8835, <0.0001, 0.0081, <0.0001, 0.0604. For $F^{1.48}A$, from left to right, p<0.0001, 0.0056, <0.0001, 0.0019, <0.0001, <0.0001. For $N^{7.49}A$, from left to right, p<0.0001, 0.0306, 0.0697, 0.0088, <0.0001, <0.0001. For $Y^{7.53}A$, from left to right, p<0.0001, 0.0371, <0.0001, <0.0001, <0.0001, 0.0002. For $F^{8.50}A$, from left to right, p<0.0001, 0.0342, <0.0001, 0.0011, <0.0001, <0.0001).

REFERENCES

1. Cao, C. *et al.* Structural basis for signal recognition and transduction by platelet-activating-factor receptor. *Nat. Struct. Mol. Biol.* **25**, 488–495 (2018).
2. Zhang, K. *et al.* Structure of the human P2Y12 receptor in complex with an antithrombotic drug. *Nature* **509**, 115–118 (2014).
3. García-Nafría, J., Nehmé, R., Edwards, P. C. & Tate, C. G. Cryo-EM structure of the serotonin 5-HT(1B) receptor coupled to heterotrimeric G(o). *Nature* **558**, 620–623 (2018).
4. Swope, W. C., Pitera, J. W. & Suits, F. Describing Protein Folding Kinetics by Molecular Dynamics Simulations. 1. Theory. *J. Phys. Chem. B* **108**, 6571–6581 (2004).
5. Husic, B. E. & Pande, V. S. Markov State Models: From an Art to a Science. *J. Am. Chem. Soc.* **140**, 2386–2396 (2018).
6. Prinz, J.-H. *et al.* Markov models of molecular kinetics: generation and validation. *J. Chem. Phys.* **134**, 174105 (2011).
7. Scherer, M. K. *et al.* PyEMMA 2: A Software Package for Estimation, Validation, and Analysis of Markov Models. *J. Chem. Theory Comput.* **11**, 5525–5542 (2015).
8. Floyd, R. W. Algorithm 97: shortest path. *Commun. ACM* **5**, 345–345 (1962).
9. Girvan, M. & Newman, M. E. J. Community structure in social and biological networks. *Proc. Natl. Acad. Sci. U. S. A.* **99**, 7821–7826 (2002).
10. Vanommeslaeghe, K. *et al.* CHARMM general force field: A force field for drug-like molecules compatible with the CHARMM all-atom additive biological force fields. *J. Comput. Chem.* **31**, 671–690 (2010).
11. Miao, Y. & McCammon, J. A. Graded activation and free energy landscapes of a muscarinic G-protein–coupled receptor. *Proc. Natl. Acad. Sci. U. S. A.* **113**, 12162–12167 (2016).
12. Miao, Y. & McCammon, J. A. Mechanism of the G-protein mimetic nanobody binding to a muscarinic G-protein-coupled receptor. *Proc. Natl. Acad. Sci. U.*

S. A. **115**, 3036–3041 (2018).

13. Roe, D. R. & Cheatham III, T. E. PTRAJ and CPPTRAJ: software for processing and analysis of molecular dynamics trajectory data. *J. Chem. Theory Comput.* **9**, 3084–3095 (2013).
14. Naritomi, Y. & Fuchigami, S. Slow dynamics in protein fluctuations revealed by time-structure based independent component analysis: the case of domain motions. *J. Chem. Phys.* **134**, 065101 (2011).
15. Suomivuori, C.-M. *et al.* Molecular mechanism of biased signaling in a prototypical G protein-coupled receptor. *Science* **367**, 881–887 (2020).
16. Maeda, S., Qu, Q., Robertson, M. J., Skiniotis, G. & Kobilka, B. K. Structures of the M1 and M2 muscarinic acetylcholine receptor/G-protein complexes. *Science* **364**, 552–557 (2019).
17. Kang, Y. *et al.* Crystal structure of rhodopsin bound to arrestin by femtosecond X-ray laser. *Nature* **523**, 561–567 (2015).
18. Le Guilloux, V., Schmidtke, P. & Tuffery, P. Fpocket: an open source platform for ligand pocket detection. *BMC Bioinformatics* **10**, 168 (2009).
19. Guarnera, E., Tan, Z. W., Zheng, Z. & Berezovsky, I. N. AlloSigMA: allosteric signaling and mutation analysis server. *Bioinformatics* **33**, 3996–3998 (2017).
20. Tan, Z. W., Guarnera, E., Tee, W.-V. & Berezovsky, I. N. AlloSigMA 2: paving the way to designing allosteric effectors and to exploring allosteric effects of mutations. *Nucleic Acids Res.* **48**, 116–124 (2020).
