# Supplementary material for: Activation pathway of a G protein-coupled receptor uncovers conformational intermediates as targets for allosteric drug design
Source: Nat Commun. 2021 Aug 5;12:4721. doi: 10.1038/s41467-021-25020-9 (PMC8342441; doi:10.1038/s41467-021-25020-9)
Supplement: Supplementary file 3 — Description of Additional Supplementary Files [file 41467_2021_25020_MOESM3_ESM.pdf]

Description of Additional Supplementary Files

File Name: Supplementary Data 1
Description: Sequences of all the primers used for site-directed mutagenesis in this study.

File Name: Supplementary Data 2
Description: Summary of current reported class A GPCR (Data were collected at April 26, 2021).
