# Supplementary material for: Activation pathway of a G protein-coupled receptor uncovers conformational intermediates as targets for allosteric drug design
Source: Nat Commun. 2021 Aug 5;12:4721. doi: 10.1038/s41467-021-25020-9 (PMC8342441; doi:10.1038/s41467-021-25020-9)
Supplement: Supplementary file 6 — Reporting Summary [file 41467_2021_25020_MOESM6_ESM.pdf]

nature portfolio

Corresponding author(s): Shaoyong Lu, Jian Zhang, Jinpeng Sun

Last updated by author(s): Jul 12, 2021

Reporting Summary

Nature Portfolio wishes to improve the reproducibility of the work that we publish. This form provides structure for consistency and transparency in reporting. For further information on Nature Portfolio policies, see our Editorial Policies and the Editorial Policy Checklist.

Statistics

For all statistical analyses, confirm that the following items are present in the figure legend, table legend, main text, or Methods section.

n/a	Confirmed	
☐	☒	The exact sample size (*n*) for each experimental group/condition, given as a discrete number and unit of measurement
☐	☒	A statement on whether measurements were taken from distinct samples or whether the same sample was measured repeatedly
☐	☒	The statistical test(s) used AND whether they are one- or two-sided<br>*Only common tests should be described solely by name; describe more complex techniques in the Methods section.*
☒	☐	A description of all covariates tested
☒	☐	A description of any assumptions or corrections, such as tests of normality and adjustment for multiple comparisons
☐	☒	A full description of the statistical parameters including central tendency (e.g. means) or other basic estimates (e.g. regression coefficient) AND variation (e.g. standard deviation) or associated estimates of uncertainty (e.g. confidence intervals)
☐	☒	For null hypothesis testing, the test statistic (e.g. *F*, *t*, *r*) with confidence intervals, effect sizes, degrees of freedom and *P* value noted<br>*Give P values as exact values whenever suitable.*
☒	☐	For Bayesian analysis, information on the choice of priors and Markov chain Monte Carlo settings
☒	☐	For hierarchical and complex designs, identification of the appropriate level for tests and full reporting of outcomes
☒	☐	Estimates of effect sizes (e.g. Cohen's *d*, Pearson's *r*), indicating how they were calculated

Our web collection on statistics for biologists contains articles on many of the points above.

Software and code

Policy information about availability of computer code

Data collection	The membrane of proteins was constructed by CHARMM-GUI2.0. Data collection for MD simulation was based on AMBER18, pmemd.cuda program with AMBER18 built-in Particle mesh Ewald method, SHAKE algorithm, and Langevin Thermostat. Molecular docking was performed by Molecular Operating Environment (MOE2019.0102) and glide algorithm in Maestro11.1, Schrödinger2017. Structure-Based Statistical Mechanical Model of Allostery was constructed by AlloSigMA 2.
Data analysis	Data analyses were based on CPPTRAJ in AMBER18 suite, VMD 1.9.2, PyEMMA 2.5.7, PyMOL2.4.0 , OriginPro 2017, MATLAB 2019a, and fpocket 3.0. Analysis of cell experiment results was performed using GraphPad Prism8.

For manuscripts utilizing custom algorithms or software that are central to the research but not yet described in published literature, software must be made available to editors and reviewers. We strongly encourage code deposition in a community repository (e.g. GitHub). See the Nature Portfolio guidelines for submitting code & software for further information.

Data

Policy information about availability of data

All manuscripts must include a data availability statement. This statement should provide the following information, where applicable:

- Accession codes, unique identifiers, or web links for publicly available datasets
- A description of any restrictions on data availability
- For clinical datasets or third party data, please ensure that the statement adheres to our policy

Relevant data supporting the findings of this study are available in the source data file provided with this paper. The activation parameter and tICA data generated in this study are provided in the Source Data file. Initial structures for MD simulation were obtained from the RCSB PDB database (PDB ID: 4YAY and 6DO1). Other GPCRs were also downloaded from the RCSB PDB database, https://www.rcsb.org/. The analysis protocol for Markov State Model referred to http://www.emma-

project.org/latest/. The network analysis in Section 9, SI was provided by http://www.scs.illinois.edu/schulten/tutorials/network/. Pocket prediction was accomplished by fpocket, see http://fpocket.sourceforge.net/. Other simulation analyses were based on AMBER suite, according to http://ambermd.org/.

Field-specific reporting

Please select the one below that is the best fit for your research. If you are not sure, read the appropriate sections before making your selection.

☒ Life sciences ☐ Behavioural & social sciences ☐ Ecological, evolutionary & environmental sciences

For a reference copy of the document with all sections, see nature.com/documents/nr-reporting-summary-flat.pdf

Life sciences study design

All studies must disclose on these points even when the disclosure is negative.

Sample size	The chosen of sample size for MD simulations refers to the timescale of GPCR activation (μs level). In Section 1, SI, the simulation system has been converged in both round number and simulation time. Thus, the sample size has been enough for research.<br>Sample sizes were not predetermined by statistical methods. For cell experiments, including G protein BRET assay, arrestin recruitment assay, FlAsH-BRET assay and ELISA assay, three independent experiments (n=3) were performed as indicated in the related figure legends. Data were analyzed by fitting various ligand concentrations and readouts using appropriate equations in Graphpad Prism software.
Data exclusions	No data were excluded.
Replication	10 rounds of simulations (300 μs in total) were applied to confirm our conclusions. All attempts of replications were successful.<br>All attempts at replication of cell experiments succeeded. Each experiment was reproduced at least three times independently as described in related Figure legends.
Randomization	In MD simulation, each run has a random velocity to start, which maintains the randomness.<br>For cell experiments, no randomization was attempted or needed. There is no animal experiment that is dependent on randomization. All variables in the present study could be controlled.
Blinding	For cell experiments, blinding was not performed as samples were allocated into pre-specified groups, and readouts were automated (e.g. plate reader). All data acquired in this study are included in this article, and subjected to statistical analysis whenever necessary.

Reporting for specific materials, systems and methods

We require information from authors about some types of materials, experimental systems and methods used in many studies. Here, indicate whether each material, system or method listed is relevant to your study. If you are not sure if a list item applies to your research, read the appropriate section before selecting a response.

Materials & experimental systems

n/a	Involved in the study	
☐	☒	Antibodies
☐	☒	Eukaryotic cell lines
☒	☐	Palaeontology and archaeology
☒	☐	Animals and other organisms
☒	☐	Human research participants
☒	☐	Clinical data
☒	☐	Dual use research of concern

Methods

n/a	Involved in the study	
☒	☐	ChIP-seq
☒	☐	Flow cytometry
☒	☐	MRI-based neuroimaging

Antibodies

Antibodies used	Monoclonal anti-FLAG (Sigma Aldrich, Cat# F1804), dilution: 1:1000<br>Goat anti-Mouse secondary antibody Thermo Fisher, Cat# A-21235), dilution: 1:5000
Validation	The antibodies used in the present study are commercially purchased and have been validated by the vendors. The antibodies were applied according to data sheet information details.Monoclonal anti-FLAG: https://www.sigmaaldrich.com/catalog/product/sigma/f1804;Goat anti-Mouse secondary antibody: https://www.thermofisher.com/cn/zh/antibody/product/Goat-anti-Mouse-IgG-H-L-Cross-Adsorbed-Secondary-Antibody-Polyclonal/A-21235

Eukaryotic cell lines

Policy information about cell lines

Cell line source(s)	HEK293 cells were obtained from American Type Culture Collection (ATCC, Manassas, VA, USA).
Authentication	All of the cell lines are maintained by the supplier. No additional authentication was performed in this study.
Mycoplasma contamination	All the cell lines were routinely tested and confirmed negative for mycoplasma contamination.
Commonly misidentified lines (See ICLAC register)	No commonly misidentified cell lines were used.
